# Automated high-throughput dispersive liquid-liquid microextraction coupled with UHPLC-MS/MS for detecting triazole fungicides in water, juices, wine, and tea

**DOI:** 10.1016/j.fochx.2025.102712

**Published:** 2025-06-30

**Authors:** Yuxin Wang, Jin Liu, Suzhen Li, Jizhen Fu, Xiaowen Wang, Li Li, Xu Jing

**Affiliations:** a College of Food Science and Engineering, Shanxi Agricultural University, Taiyuan, Shanxi 030031, China,; b Shanxi Key Laboratory of Integrated Pest Management in Agriculture, College of Plant Protection, Shanxi Agricultural University, Taiyuan, Shanxi 030031, China,

**Keywords:** Liquid phase microextraction, Solvents, Food safety, Automation, Fatty acids

## Abstract

The widespread use of triazole fungicides has raised concerns over residual. This study presents an automated, environmentally friendly, simple, and efficient method for detecting triazole fungicides in food samples, utilizing high-throughput liquid processing, dispersive liquid-liquid microextraction, and ultra-high-performance liquid chromatography-tandem mass spectrometry. The automated liquid handling workstation was employed to automate the whole sample pre-treatment process. Fatty acid, bio-based solvent, and saturated sodium chloride solution were used as the extractant, dispersant, and demulsifier, respectively. The whole operation process obviated the use of toxic reagents and eliminated the need for time-consuming centrifugation. The linear ranged from 0.01 to 1 μg L^−1^with limits of detection and quantification of 0.003 μg L^−1^ and 0.01 μg L^−1^, respectively. Ten triazole fungicides were successfully detected in food samples with recoveries of 70.1 %–105.7 %. The automation of dispersive liquid-liquid microextraction demonstrates huge potential for broad applicability within the field of food safety testing.

## Highlights

• The automated pre-treatment technique minimizes manual operation errors.
• Four samples can be processed simultaneously, increasing analytical throughput.
• Bio-based solvents are employed as dispersants instead of toxic alternatives.
• Ten TFs are detected within 5.5 min using UHPLC-MS/MS.
• An eco-friendly, simple, and efficient method is developed for food sample analysis.

## 1 Introduction

Triazole fungicides (TFs) have been the most extensively used fungicide class globally since the 1970s due to their broad-spectrum and highly effective antifungal properties (Cai et al., 2025). They currently constitute more than 23 % of the global fungicide market (Pimentão et al., 2024). To safeguard public health, the Codex Alimentarius Commission has established a stringent maximum residue limit of 0.01 mg kg^−1^ for TFs in food products (Kachangoon, Santaladchaiyakit, et al., 2024). Extensive research has demonstrated that TFs exhibit significant toxicity even at trace concentrations, raising substantial concerns regarding their mutagenic and carcinogenic potential (Eissa et al., 2024). These environmentally persistent compounds possess long half-lives ranging from several weeks to months. Improper agricultural practices, including excessive application or inappropriate usage, contribute to an increase in these residue risks (Xing et al., 2023). The issue of pesticide residues poses a significant challenge to food safety. Accordingly, it is essential to develop a sustainable, simple, and efficient approach for detecting TFs in food to ensure compliance with regulatory limits. Given the interference from complex matrices and the trace residue characteristics of TFs, an appropriate sample pre-treatment step is essential to extract target analytes.

In recent years, various pre-treatment techniques have been developed to extract TFs. Liquid-liquid extraction technology is a conventional pre-treatment technique. However, its substantial consumption of organic solvents, reliance on toxic reagents, and labor-intensive procedures limit its compatibility with the principles of green analytical chemistry (Płotka-Wasylka et al., 2016). To overcome these drawbacks, dispersive liquid-liquid microextraction (DLLME) has emerged as an alternative, exhibiting advantages including low solvent usage, high extraction efficiency, cost-effectiveness, and miniaturization, and is gradually replacing traditional liquid-liquid extraction methods (Xie et al., 2023). DLLME typically involves the rapid injection of a mixture comprising a water-immiscible extractant and a water-miscible dispersant into samples with analytes, forming a turbid solution that facilitates efficient extraction (Daghi et al., 2022). Nevertheless, conventional DLLME techniques still present critical limitations, especially the use of toxic organic solvents as both extractants and dispersants.

DLLME typically uses halogenated solvents, including chloroform and carbon tetrachloride, as extractants (Pirsaheb et al., 2013). Although such solvents are favorable for extraction operations, their inherent toxicity and bioaccumulation endanger both experimental personnel and ecosystems. In recent years, deep eutectic solvents (DESs) have attracted attention in the field of DLLME as green solvents due to their environmental friendliness, low toxicity, and biodegradability (Fattahi et al., 2024). They are formed through hydrogen bonding between donors and acceptors under heating (Hassanpour et al., 2023; Zhang, Zhang, et al., 2023). However, DESs face challenges, including the requirement for specific preparation steps, precise control of component ratios, time-consuming synthesis, and potential high viscosity that may compromise extraction efficiency. To address these limitations, this study utilizes only pure fatty acids as an extractant, retaining environmental advantages while simplifying solvent preparation and enhancing methodological practicality. Fatty acids, which are naturally occurring bioactive components in mammalian milk, have become a class of promising alternatives to conventional toxic solvents due to their excellent biodegradability, renewable properties, and compatibility with a wide range of analytical techniques (Jing et al., 2021; Yuan et al., 2025).

Traditional DLLME relies on highly toxic dispersants such as methanol, acetonitrile, and acetone, which contradict the principles of green analytical chemistry. Dispersants play a crucial role in promoting the formation of fine droplets of hydrophobic extractants and ensuring their uniform distribution within the aqueous phase. Over the past few years, bio-based solvents such as γ-valerolactone (GVL), diesters (DBE), and dimethyl carbonate (DMC) have emerged as new green solvents, demonstrating huge potential as dispersants in DLLME due to their unique physicochemical properties. Specifically, GVL, a typical product derived from the conversion of lignocellulosic biomass, offers an environmentally benign alternative, effectively reducing the toxicity risks associated with conventional solvents (Raj et al., 2021). DBE, synthesized via the esterification of dicarboxylic acids and alcohols, is recognized as a low-toxicity, non-carcinogenic, and non-corrosive solvent (Koyama et al., 2009). DMC which can be sustainably synthesized through catalytic conversion of industrial waste gases or CO_2_ is characterized by low toxicity and rapid biodegradability (Patraşcu et al., 2022).

Furthermore, auxiliary devices such as vortex mixers, ultrasonic baths, and mechanical shakers are commonly utilized to improve the efficiency of the DLLME process. However, these methods not only necessitate additional specialized equipment but also often lead to inconsistent energy during mixing, potentially causing localized overmixing or undermixing (Sajid, 2022). Automation and high-throughput technologies have emerged as key advancements in sample pretreatment techniques. Herein, an automated liquid-handling workstation is introduced to facilitate the extraction process through continuous pumping operations. It is well-established that automated liquid handling workstations can precisely manage the transfer of samples, extractants, dispersants, and emulsifiers between deep-well plates and liquid reservoirs, all under programmed control (Ju et al., 2025). Accordingly, the development of automated high-throughput DLLME may not only reduce the labor intensity of laboratory personnel but also minimize human errors, significantly improving the efficiency of sample processing and ensuring high repeatability of the experimental process.

Conventional DLLME technology requires centrifugation for phase separation, which prolongs the sample pre-treatment time. To overcome this limitation, recent research has demonstrated that the introduction of magnetic field can effectively achieve rapid separation of aqueous and magnetic organic phases, thereby avoiding traditional centrifugation operations (Mohebbi et al., 2023). This study proposes an improved method based on the salting-out effect is proposed. By adding a saturated sodium chloride solution as a demulsifier to the extractant-dispersant-water system, rapid emulsion destabilization is achieved, allowing for rapid phase separation and reduced separation time (Xia et al., 2012).

This study presents an automated high-throughput DLLME method coupled with ultra-high-performance liquid chromatography-tandem mass spectrometry (UHPLC-MS/MS) to determine TFs in food matrices. Natural octanoic acid as the extractant and bio-based GVL as the dispersant were employed in this method, aligning with the tenets of green chemistry by circumventing the use of conventional toxic reagents. The automation of the process was achieved through an eight-channel automatic liquid handling workstation. This workstation orchestrates the sequential steps of tip loading, sample introduction, solvent mixing, dispersion, and tip unloading in a synchronized manner. As a result, it allowed for the simultaneous processing of four samples. This capability not only enhanced analytical efficiency but also substantially minimized human errors. Instead of relying on the traditional centrifugation step for phase separation, a saturated sodium chloride solution was utilized. This innovation led to a significant reduction in phase separation time. Furthermore, rapid detection of ten TFs was achieved within 5.5 min using UHPLC-MS/MS. The green solvent-based automated high-throughput DLLME technology demonstrates huge potential for the efficient analysis of ten TFs in food matrices using UHPLC-MS/MS.

## 2 Materials and methods

### 2.1 Reagents and chemicals

Mefentrifluconazole, propiconazole, tebuconazole, bitertanol, metconazole, triadimefon, epoxiconazole, hexaconazole, myclobutanil, difenoconazole, hexanoic acid, heptanoic acid, octanoic acid, nonanoic acid, GVL, DME, DMC, ammonium formate, formic acid and methanol were purchased from Aladdin Co., Ltd. (Guangzhou, China). Commercially available beverage samples (drinking water, fruit juice, green tea, and wine) were obtained from local markets (Taiyuan, China).

### 2.2 UHPLC-MS/MS analysis

The samples were analyzed using a Nexera LC-40 ultra-high performance liquid chromatography system (Shimadzu Corporation, Japan) coupled to a SCIEX 4500 triple quadrupole mass spectrometer (AB Sciex Corporation, USA). Ten TFs were completely detected within 5.5 min employing a Waters ACQUITY UPLC BEH C18 column (2.1 mm × 100 mm, 1.7 μm) at 40 °C with an injection volume of 2 μL. Gradient elution was performed using a mobile phase consisting of 0.01 % aqueous formic acid containing 2 mmol L^−1^ ammonium formate (A) and methanol (B). The mobile phase composition was varied as follows: 0–0.5 min, 5 % B; 1.3–4.5 min, 80 % B; 4.6–5.5 min, 5 % B. The optimal mass spectrometry parameters for the ten TFs were acquired in full scan mode under positive ionization using multiple reaction monitoring (MRM), with detailed parameters provided in Table S1.

### 2.3 Automated high-throughput DLLME procedure

**Fig. 1:**
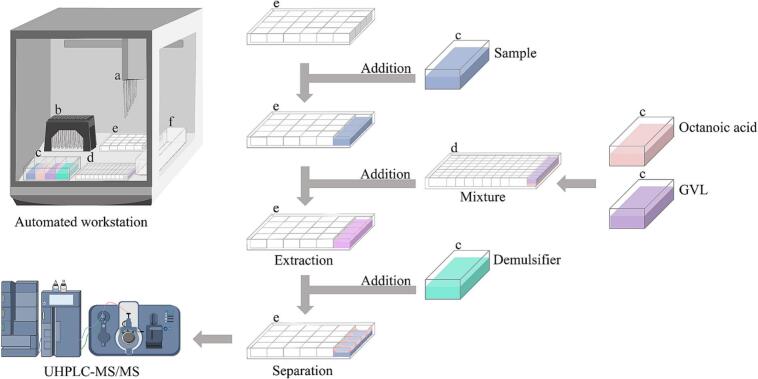
Procedure for DLLME-UHPLC-MS/MS analysis.

The automated liquid handling workstation integrates seven key components: a control panel, a pipetting robotic arm (a), a tip box (b), a reagent reservoir (c), a 96 deep-well plate (d, 4 × 6 configuration, 10 mL/well), a 24 deep-well plate (e, 8 × 12 configuration, 1.2 mL/well), and a waste reservoir (f). The detailed operational steps are illustrated in Fig. 1.

The eight-channel pipetting robotic arm first loaded tips at component b, then aspirated four 5 mL samples from component c and dispensed them into a 24-well plate at component e before unloading the tips at component f.

Next, the pipette tips aspirated four equal volumes of extractant (octanoic acid, 183 μL each) from the extractant slot at component c and dispensed it into a 96-well deep-well plate at component d. The tips were then unloaded again at component f.

Subsequently, the pipette tips aspirated four equal volumes of dispersant (GVL, 367 μL each) from the dispersant slot at component c and dispensed it into the 96-well deep-well plate at component d.

The mixture was then transferred to the 24-well deep-well plate at component e containing 5 mL of the sample and mixed for 7 cycles. After completing this step, the arm moved to position f to unload the tips.

Finally, the pipette tips aspirated four equal volumes of demulsifier (saturated sodium chloride solution, 200 μL each) from the demulsifier slot at component c and dispensed it into the 24-well deep-well plate at component e, which contained samples to initiate demulsification.

The 24-deep well plate enables parallel processing of four independent samples simultaneously using the eight-channel pipetting system.

After allowing the mixture to stand for 5 min to complete the separation, 20 μL of the octanoic acid was aspirated from each well in the 24-well deep-well plate for UHPLC-MS/MS analysis.

## 3 Results and discussion

### 3.1 Optimization of automated high-throughput DLLME conditions

To achieve efficient extraction of ten TFs from samples, a systematic optimization of seven critical factors in the DLLME procedure was conducted. These factors included the type of extractant and dispersant, the volume ratio of extractant to dispersant, the total volume of extractant and dispersant, the number of mixing cycles, the mixing speed, and the volume of demulsifier. All factors were assessed using triplicate independent replicates, with the optimization assessed based on the mean recovery and relative standard deviations (RSDs) to identify the optimal conditions. Through preliminary experiments, the fixed and invariant parameter combinations of each factor were determined as follows: octanoic acid as the extractant, GVL as the dispersant, a volume ratio of extractant to dispersant of 1:2, a total volume of dispersant and extractant of 550 μL, the number of 7 mixing cycles, a mixing speed of 610 μL s^−1^, and a demulsifier volume of 200 μL.

#### 3.1.1 Influence of the extractant type

**Fig. 2:**
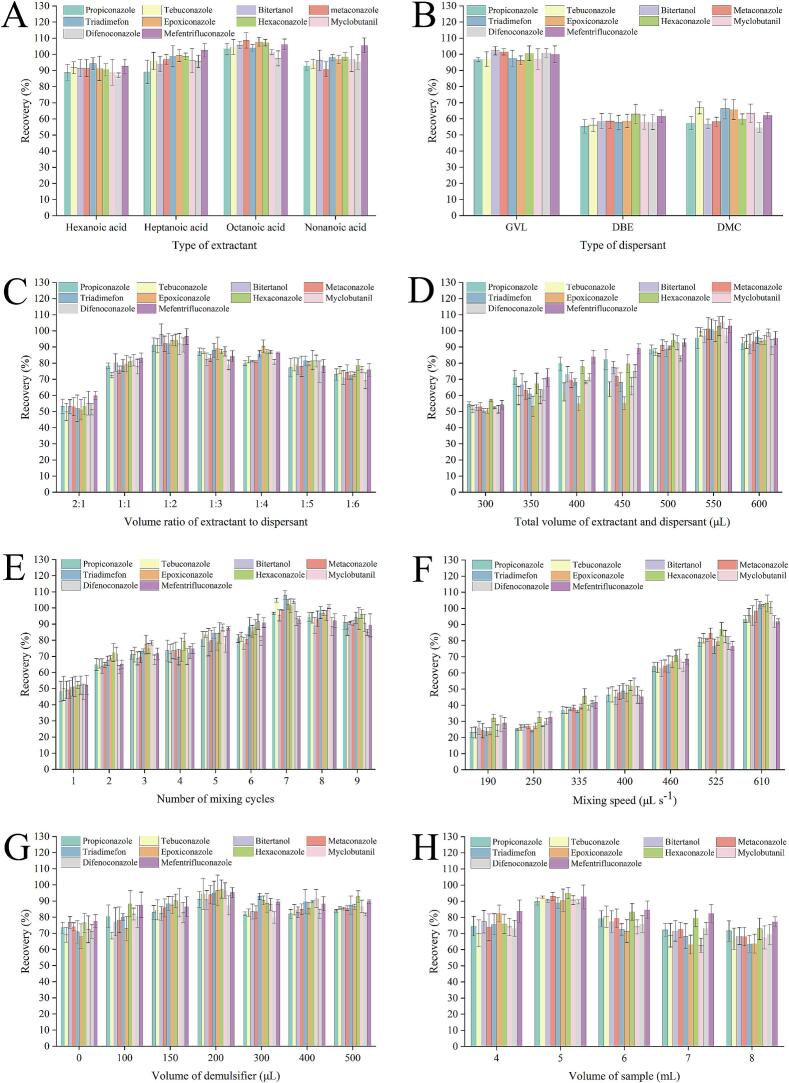
Influence of the DLLME method. Type of extractant (A); type of dispersant (B); volume ratio of extractant to dispersant (C); total volume of extractant and dispersant (D); number of mixing cycles (E); mixing speed (F); volume of demulsifier (G); and volume of sample (H).

The selection of an appropriate extractant is crucial for optimal extraction efficiency in DLLME, as the affinity of different extractants for target analytes varies significantly (El-Deen & Shimizu, 2019). An ideal extractant should possess low volatility, low density, and immiscibility with water. To evaluate the impact of different extractants on the extraction performance of TFs, the effects of four fatty acids (hexanoic, heptanoic, octanoic, and nonanoic acid) on the recovery of TFs were investigated. All four types of fatty acids met the essential criteria for use as extractants. As shown in Fig. 2A, octanoic acid demonstrated the highest recoveries, attributed to the moderate hydrophobicity of octanoic acid, whose molecular structure promotes favorable interactions with TFs through synergistic hydrophobic and hydrogen bonding, thereby enhancing extraction efficiency. Therefore, octanoic acid was chosen as the optimal extractant for further investigation.

#### 3.1.2 Influence of the dispersant type

An effective dispersant promotes the uniform dispersion of the extractant into fine droplets, enhances the interfacial surface area, and increases the mass transfer between the extractant and water phases, thereby improving the recovery of target analytes. The selection of bio-based solvents, including GVL, DBE, and DMC, as dispersants, was optimized in this study, given their environmental friendliness, low toxicity, and biodegradability, in comparison with traditional solvents such as acetonitrile, methanol, and acetone. As shown in Fig. 2B, GVL achieved the highest recovery. As a cyclic ester, GVL contained a lactone ring. In contrast, DBE and DMC with linear ester structure resulted in poor dispersion of extractants within the aqueous phase (Folprechtová et al., 2024; Hou et al., 2025). Therefore, in subsequent experiments, GVL was chosen as the dispersant.

#### 3.1.3 Influence of the volume ratio of extractant to dispersant

The recovery of target analytes is influenced by the ratio of extractant to dispersant, as it affects droplet formation and the dispersion efficiency of extractants. To determine the optimal volume ratio, seven different volume ratios (2:1, 1:1, 1:2, 1:3, 1:4, 1:5, and 1:6) were investigated in this experiment. As illustrated in Fig. 2C, the recovery of TFs initially increased before declining. Excessive GVL may lead to partial dissolution of octanoic acid in the aqueous phase, resulting in reduced efficiency. Consequently, a 1:2 volume ratio of extractant to dispersant was chosen as the optimal volume ratio for subsequent experiments.

#### 3.1.4 Influence of the total volume of extractant and dispersant

An optimal total volume of extractants and dispersants not only ensures efficient extraction (Mousa et al., 2025), but also minimizes solvent consumption, promotes environmental sustainability, and conforms to the principles of green chemistry (Custodio-Mendoza et al., 2024). To determine the optimal total volume of extractants and dispersants, seven different volumes (300, 350, 400, 450, 500, 550, and 600 μL) were investigated in this experiment. As illustrated in Fig. 2D, the recovery of ten TFs initially increased and then decreased as the total volume varied from 300 to 600 μL, peaking at 550 μL. During the ascending stage, the enhanced recovery was attributed to the initially insufficient total volume, leading to inadequate distribution of TFs. Conversely, the decline in recovery during the descending phase resulted from excessive dissolution of octanoic acid and GVL, increasing the solubility of TFs in samples. Therefore, a total volume of 550 μL of extractant and dispersant was determined as the most effective volume for subsequent experiments.

#### 3.1.5 Influence of the number of mixing cycles

Repeated aspiration and mixing can enhance the uniform dispersion of extractants in the sample system, resulting in the formation of smaller droplets of extractants and increasing the contact area between the two phases (Magdy Saad et al., 2024). To determine the optimal number of cycles, nine different cycles (1, 2, 3, 4, 5, 6, 7, 8, and 9) were investigated in this experiment. As illustrated in Fig. 2E, the recovery of TFs first increased and then decreased with an increasing number of cycles, reaching its peak at 7 cycles. The observed trend may be due to incomplete dispersion of octanoic acid within the sample matrix when the number of mixing cycles was inadequate, leading to incomplete transfer of TFs. However, an excessive number of mixing cycles can form overly stable dispersed droplets, reducing the demulsification efficiency and ultimately affecting the phase separation effect. Therefore, 7 cycles were identified as the optimal number of cycles for further studies.

#### 3.1.6 Influence of the mixing speed

The boundary layer, a critical region for mass transfer between the sample and the extractant phases, directly influences the dispersion effectiveness of octanoic acid and the mass transfer efficiency of TFs. The mixing speed further modulates the mass transfer resistance by altering the thickness of the boundary layer. To determine the optimal mixing speed, nine different mixing speeds (190, 250, 335, 400, 460, 525, and 610 μL s^−1^) were investigated in this experiment. As illustrated in Fig. 2F, the recovery consistently increased with increased mixing speed, reaching its peak value at 610 μL s^−1^. Accordingly, 610 μL s^−1^ was selected as the optimal mixing speed for subsequent experiments.

#### 3.1.7 Influence of the volume of demulsifier

During demulsification, electrolyte solutions such as sodium chloride facilitate the prompt and efficient isolation of extractant droplets by modulating the ionic strength (Guo et al., 2024). However, both insufficient and excessive amounts can adversely affect the droplet separation efficiency and reduce the recovery of target analytes. To determine the optimal volume of demulsifier, seven different volumes (0, 100, 150, 200, 300, 400, and 500 μL) were investigated in this experiment. As shown in Fig. 2G, the recovery of TFs increased initially and then decreased as the volume of demulsifier increased from 0 to 500 μL, reaching its peak at 200 μL. This trend can be ascribed to the fact that an insufficient volume of demulsifier fails to adequately disrupt the emulsion, leading to incomplete droplet separation and the absence of a distinct two-phase system, ultimately reducing the recovery. Conversely, an excessive amount of demulsifier can significantly enhance the viscosity of the sample solution, thereby hindering the mass transfer of TFs and reducing the extraction efficiency. Accordingly, a demulsifier volume of 200 μL was determined as the optimal volume for the following experiments.

#### 3.1.8 Influence of volume of sample

An appropriate sample volume can improve both the sensitivity and enrichment capability of the DLLME method (Soylak et al., 2025). To determine the optimal sample volume, five different volumes (4, 5, 6, 7, and 8 mL) were investigated in this experiment. As illustrated in Fig. 2H, a sample volume exceeding 5 mL led to decreased TF recovery, attributed to the dilution effect, causing a relatively inadequate volume of octanoic acid, which subsequently hindered the thorough transfer of TFs, ultimately resulting in decreased recovery. Therefore, 5 mL was determined to be the optimal sample volume for the following experiments.

### 3.2 Characterization of the emulsion

The emulsion in the extraction process was evaluated by stereo microscopic observation and zeta potential measurement. A mixed solution of extractant and dispersant was injected into the sample and mixed for 7 cycles using an automated pipette to ensure system uniformity. The emulsion was aspirated in small aliquots using a micropipette and deposited at the center of a clean glass slide, followed by careful placement of a coverslip to avoid bubble formation. Finally, the dispersion of the emulsion was observed at magnifications of 40 X, 80 X, and 135 X under a stereo microscope, respectively. The experimental results (Fig. S1) showed that the emulsion was nearly spherical and uniformly distributed, confirming that the extractant was well dispersed in the sample.

Zeta potential is a key indicator for characterizing the dispersion state of extractants, and the stability of the system is evaluated by measuring the surface charge of droplets in the three-phase system (extractant-dispersant-sample). Three parallel measurements were conducted to ensure data reliability, and the average zeta potential was measured to be −23.2 mV (| ζ | =23.2 mV). According to the theory of colloidal stability, the system exhibits good electrostatic stability when | ζ | > 20 mV (Yang et al., 2025). The experimental results (Fig. S2) satisfied the specified criterion, indicating that the extractant droplets were uniformly dispersed and demonstrated a low aggregation propensity, ultimately forming a stable emulsion system.

### 3.3 Method validation

Under optimal experimental conditions, the DLLME method underwent a comprehensive validation process, which included the evaluation of metrics such as the linear equation, coefficient of determination (*R*^2^), limit of detection (LOD), limit of quantification (LOQ), and intra-day and inter-day RSDs (Table 1). The validation results demonstrated that TFs exhibited an excellent linear relationship within the concentration range of 0.01 to 1 μg L^−1^, with an *R*^2^ value exceeding 0.996. Using the signal-to-noise ratio (S/N) as the measurement criterion, the LOD was determined with an S/N threshold of 3, while the LOQ was established with an S/N threshold of 10. Through precise calculations, the LOD and LOQ were determined to be 0.003 and 0.01–1, respectively. The reproducibility of the method was evaluated through intra-day and inter-day experiments, yielding RSDs in the ranges of 2.0 %–6.5 % and 2.1 %–7.5 % (*n* = 5), respectively. Taken together, these results highlight the method's exceptional sensitivity and reliability.

**Table 1:** Analytical parameters of the DLLME-UHPLC-MS/MS method.

TF	Sample	Linear equation (μg L^−1^)	*R*^2^	LOD (μg L^−1^)	LOQ (μg L^−1^)	Intra-day RSD (%)	Inter-day RSD (%)
Propiconazole	Water	*y* = 2,265,872*×* + 4524	0.999	0.003	0.01	4.1	5.8
	Juice	*y* = 2,088,689*×* + 9861	0.999	0.003	0.01	4.7	4.6
	Wine	*y* = 2,025,773*×* + 29,653	0.999	0.003	0.01	3.4	5.1
	Tea	*y* = 2,327,136*×* - 1071	0.999	0.003	0.01	3.9	3.2
Tebuconazole	Water	*y* = 945,296*×* – 341.7	0.999	0.003	0.01	5.9	5.6
	Juice	*y* = 803,176*×* + 4207	0.999	0.003	0.01	3.7	6.9
	Wine	*y* = 762,031*×* + 18,696	0.997	0.003	0.01	4.7	4.9
	Tea	*y* = 892,466*×* + 6139	0.999	0.003	0.01	2.9	5.2
Bitertanol	Water	*y* = 1,071,908*×* + 1303	0.999	0.003	0.01	4.4	4.3
	Juice	*y* = 1,033,026*×* + 3535	0.999	0.003	0.01	5.3	6.6
	Wine	*y* = 961,453*×* + 16,676	0.998	0.003	0.01	4.4	2.1
	Tea	*y* = 1,196,675*×* - 570	0.999	0.003	0.01	4.6	3.9
Metconazole	Water	*y =* 885,432*×* + 4185	0.999	0.003	0.01	3.5	5.1
	Juice	*y =* 885,432*× +* 4185	0.999	0.003	0.01	3.0	6.7
	Wine	*y =* 865,129*× +* 14,661	0.998	0.003	0.01	4.0	5.7
	Tea	*y =* 1,067,540*×* + 1917	0.999	0.003	0.01	4.7	4.2
Triadimefon	Water	*y =* 949,785*× +* 19,001	0.998	0.003	0.01	3.0	5.0
	Juice	*y =* 769,053*×* + 18,514	0.997	0.003	0.01	6.5	6.3
	Wine	*y* = 817,805*×* + 15,917	0.999	0.003	0.01	5.5	5.3
	Tea	*y* = 924,327*×* + 12,349	0.998	0.003	0.01	3.9	3.6
Epoxiconazole	Water	*y* = 582,799*×* + 8025	0.998	0.003	0.01	3.9	5.7
	Juice	*y* = 486,929*×* + 17,555	0.999	0.003	0.01	4.3	7.0
	Wine	*y* = 519,512*×* + 7817	0.999	0.003	0.01	4.0	4.0
	Tea	*y* = 579,759*×* + 7521	0.999	0.003	0.01	3.6	4.0
Hexaconazole	Water	*y* = 493,113*×* + 1652	0.999	0.003	0.01	3.5	5.5
	Juice	*y* = 443,825*×* + 3151	0.999	0.003	0.01	4.7	7.1
	Wine	*y* = 432,871*×* + 11,839	0.996	0.003	0.01	3.2	3.8
	Tea	*y* = 495,871*×* + 2286	0.999	0.003	0.01	3.4	4.4
Myclobutanil	Water	*y* = 544,001*×* + 12,695	0.999	0.003	0.01	4.3	3.6
	Juice	*y* = 432,354*×* + 17,171	0.999	0.003	0.01	5.4	3.3
	Wine	*y* = 472,448*×* + 15,943	0.998	0.003	0.01	5.0	5.2
	Tea	*y* = 496,515*×* + 16,669	0.999	0.003	0.01	5.9	5.2
Difenoconazole	Water	*y* = 1,898,344*×* + 8312	0.999	0.003	0.01	2.1	4.7
	Juice	*y* = 1,681,527*×* + 6591	0.999	0.003	0.01	3.0	4.5
	Wine	*y* = 1,767,963*×* + 18,152	0.999	0.003	0.01	2.0	3.0
	Tea	*y* = 1,944,836*×* + 5035	0.999	0.003	0.01	3.8	3.9
Mefentrifluconazole	Water	*y* = 70,053*×* + 1740	0.999	0.003	0.01	5.9	6.3
	Juice	*y* = 64,152*×* + 2032	0.998	0.003	0.01	3.0	7.5
	Wine	*y* = 61,466*×* + 2157	0.999	0.003	0.01	6.3	5.9
	Tea	*y* = 68,292*×* + 524	0.999	0.003	0.01	4.4	5.0

### 3.4 Sample analysis of water, juice, wine, and tea

To assess the applicability of the DLLME method for analyzing TFs, water, juice, wine, and tea were prepared at known concentrations of 0.01, 0.1, and 1 μg L^−1^ for subsequent analysis. Five replicate experiments were performed for each concentration. The UHPLC-MS/MS chromatograms of blank samples and TFs spiked samples was detailed in Figs. S3–6. The analysis results showed that there were no chromatographic peaks that interfered with the analysis of the TFs in the real samples. As shown in Table 2, the recovery of TFs in the food samples was within the range of 70.1 %–105.7 %, accompanied by RSDs varying from 1.0 % to 8.7 %. The recoveries and RSDs complied with the requirements of the International Food Standard CXG 90-2017 from Codex Alimentarius Commission. These findings indicate that the DLLME method offers excellent accuracy and precision, making it suitable for detecting TFs in food samples.

**Table 2:** Analysis of TFs in actual samples.

TF	Spiked concentration (μg L^−1^)	Water		Juice		Wine		Tea	
		Recovery (%)	RSD (%)	Recovery (%)	RSD (%)	Recovery (%)	RSD (%)	Recovery (%)	RSD (%)
Propiconazole	0	–	–	–	–	–	–	–	–
	0.01	88.0	5.4	83.2	8.4	80.2	4.8	73.1	3.2
	0.1	86.3	7.7	76.6	7.7	72.8	3.9	71.3	5.1
	1	92.6	5.4	73.1	6.5	77.0	5.3	71.9	5.3
Tebuconazole	0	–	–	–	–	–	–		
	0.01	88.7	8.4	88.7	3.4	78.0	7.9	78.0	4.9
	0.1	86.9	7.1	78.1	7.1	72.4	3.3	71.7	5.0
	1	96.3	5.1	74.1	7.7	81.0	5.7	72.6	4.3
Bitertanol	0	–	–	–	–	–	–		
	0.01	86.9	8.0	80.8	5.6	78.7	4.6	70.3	2.3
	0.1	83.5	6.8	74.7	6.1	71.4	1.0	70.2	3.8
	1	88.5	5.4	70.1	6.5	70.6	4.5	71.5	4.3
Metconazole	0	–	–	–	–	–	–		
	0.01	84.9	6.4	81.5	7.0	78.2	8.0	70.4	2.6
	0.1	84.8	7.7	77.6	7.4	74.7	2.3	72.3	4.5
	1	90.7	5.3	74.1	6.7	76.5	4.6	70.9	3.5
Triadimefon	0	–	–	–	–	–	–		
	0.01	98.0	5.2	87.2	3.7	77.5	5.0	77.5	5.6
	0.1	85.0	6.7	86.0	7.4	76.1	3.6	80.0	4.5
	1	92.2	6.5	77.8	8.3	94.1	8.4	76.6	4.4
Epoxiconazole	0	–	–	–	–	–	–		
	0.01	83.5	8.3	84.6	7.4	76.6	7.5	72.3	3.0
	0.1	83.3	3.7	88.4	7.4	70.4	4.1	76.6	4.8
	1	95.4	5.8	73.4	5.4	77.9	3.9	75.5	5.7
Hexaconazole	0	–	–	–	–	–	–		
	0.01	97.7	6.7	95.6	7.1	92.9	3.5	99.1	8.7
	0.1	95.9	7.0	90.8	7.4	87.6	4.2	84.2	7.6
	1	96.3	6.0	77.7	4.9	80.8	6.6	76.9	5.7
Myclobutanil	0	–	–	–	–	–	–		
	0.01	94.0	7.6	90.1	3.0	86.4	3.9	85.3	5.4
	0.1	82.4	6.4	85.8	6.8	86.8	5.5	75.0	5.2
	1	87.5	3.9	74.9	7.1	103.2	3.7	75.8	5.1
Difenoconazole	0	–	–	–	–	–	–		
	0.01	83.0	6.5	82.4	2.3	74.3	7.3	73.5	5.4
	0.1	78.7	6.4	74.7	6.3	73.6	2.3	71.3	3.5
	1	82.4	3.8	73.8	7.7	70.7	5.9	72.2	1.7
Mefentrifluconazole	0	–	–	–	–	–	–		
	0.01	105.7	5.4	90.8	7.1	93.0	6.7	86.8	5.6
	0.1	92.6	5.0	80.6	4.2	75.4	1.9	70.3	5.7
	1	95.6	6.6	72.8	6.8	83.5	4.4	72.4	4.9

### 3.5 Assessment of method greenness

The environmental friendliness of analytical techniques was systematically evaluated from various perspectives by utilizing four different green assessment tools: Complex Modified Green Analytical Procedure Index (ComplexMoGAPI), Sample Preparation Metric of Sustainability (SPMS), and Greenness evaluation metric for analytical methods (GEMAM). Moreover, the Blue Applicability Grade Index (BAGI) was utilized to systematically evaluate the applicability of the sample pre-treatment approach.

**Fig. 3:**
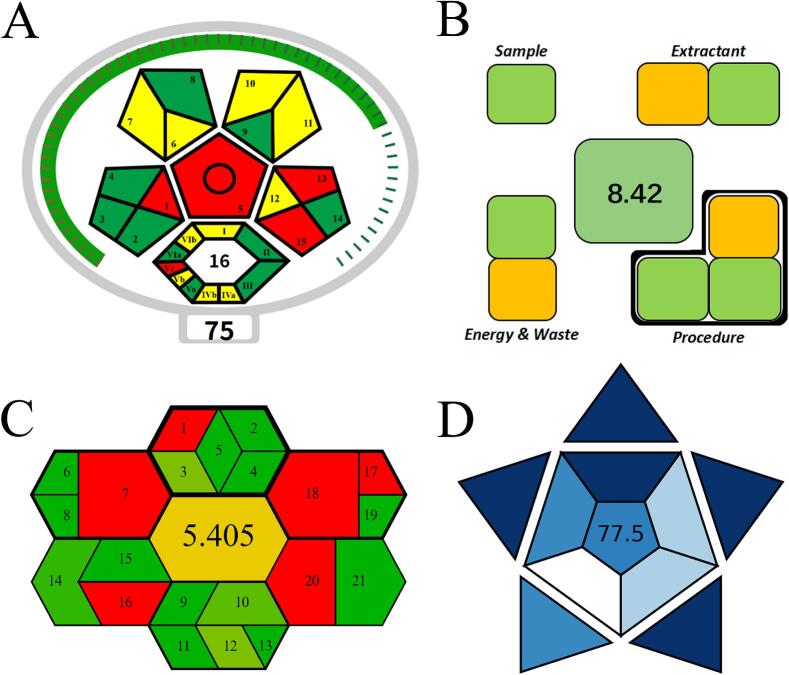
Green assessment of DLLME-UHPLC-MS/MS. ComplexMoGAPI (A); SPMS (B); GEMAM (C); and BAGI (D). (For interpretation of the references to color in this figure legend, the reader is referred to the web version of this article.)

The ComplexMoGAPI technique integrates 26 green analytical chemistry indicators to build a comprehensive analytical method assessment system (Zughaibi & Al-Asmari, 2025). The technique overcomes the limitations of traditional stepwise assessment by evaluating the health and environmental impacts of analytical methods. The ComplexMoGAPI assessment demonstrated that the DLLME method offers three key green chemistry advantages: direct use of available reagents eliminates synthetic purification requirements, complete avoidance of toxic reagents, and a significant reduction in waste generation. The result of the assessment was scored as 75 (Fig. 3A and Table S2).

The SPMS assessment system systematically evaluates the green attributes of sample preparation techniques through a combination of quantitative scoring and qualitative grading. The proposed method was assessed by SPMS with a score of 8.42/10 (Fig. 3B and Table S3).

The GEMAM system evaluates analytical methods using 12 principles of green chemistry and 10 pre-treatment metrics, giving a score of 0–10 (hexagonal pictograms) and color-coding the results (Xin et al., 2025). The method was assessed by GEMAM with a score of 5.41/10 (Fig. 3C and Table S4).

The BAGI tool assesses the suitability of the method using asteroidal pictograms with a color-coded scoring system (dark blue = 10 points, blue = 7.5 points, light blue = 5 points, white = 2.5 points) (Manousi et al., 2025). The DLLME method received a score of 77.5 (Fig. 3D and Table S5).

Based on the results of these four green assessments, it can be concluded that the developed method is an environmentally sustainable method for high-throughput trace analysis of complex matrices.

### 3.6 Density functional theory calculations

**Fig. 4:**
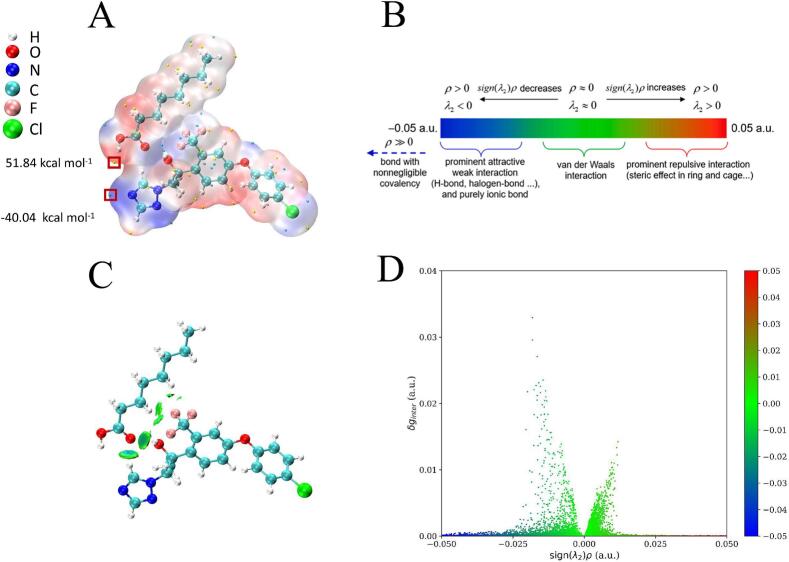
Molecular electrostatic potential diagram of octanoic acid and mefentrifluconazole (A); commonly used color scale in non-covalent interaction map and common interpretation of various color ranges (B); and the sign (λ_2_) *ρ* colored isosurfaces (C) and scatter plot (D) of IGM between octanoic acid and mefentrifluconazole.

Gaussian 16 software was used to systematically analyze the interaction mechanism between the extractant (octanoic acid) and the target analyte (mefentrifluconazole) using molecular electrostatic potential (MEP) and independent gradient model (IGM). Geometries were optimized at the B3LYP/6-31G(d) level with the incorporation of D3 dispersion correction, followed by wavefunction analysis using Multiwfn and visualization with Visual Molecular Dynamics. As shown in Fig. 4A, MEP analysis revealed significant spatial heterogeneity in the distribution of molecular surface electrostatic potential: the red region (local maximum +51.84 kcal mol^−1^) was mainly distributed around hydrogen atoms, indicating their nucleophilic properties, while the blue region (local minimum of −40.04 kcal mol^−1^) was concentrated around the carbon, nitrogen atoms in the triazole ring, reflecting electrophilic properties. The complementary distribution of this electrostatic potential provides an essential driving force for molecular recognition and binding. The color-coded IGM analysis (Fig. 4B) was used to analyze weak intra-and inter-molecular interactions, with blue, green, and red areas representing weak interactions (such as hydrogen bonds), van der Waals interactions, and repulsive interactions. Fig. 4C and D show the 3D contour map and 2D cross-section of the molecular system, respectively. In the 3D contour map (Fig. 4C), a large number of green contour surfaces were observed, indicating that van der Waals interactions constituted the predominant form of molecular binding. The pale blue iso surfaces between white and red spheres indicated weak hydrogen bonds formed by hydrogen atoms and oxygen atoms. In the 2D cross-sectional analysis diagram (Fig. 4D), two characteristic green peaks were observed, corresponding to sign (λ₂) *ρ* of −0.015 and + 0.01 a.u., respectively, which coincided with the green area in the 3D contour map (Fig. 4C). This result indicated that van der Waals interactions played a dominant role in the molecular binding process, while hydrogen bonding interactions were weak, and repulsive effects were almost non-existent. Therefore, the interaction between octanoic acid and mefentrifluconazole was mainly maintained by van der Waals forces characterized by green isosurfaces, which was in line with the results of electron density analysis.

### 3.7 Comparison with other methods

Next, a comprehensive comparison of the DLLME method was conducted with those reported within the past three years for extracting TFs from food samples, analyzing various aspects such as extraction method, automation level, sample throughput, sample, solvent, device utilized, instrument, LOD, and LOQ in Table 3. In contrast to existing methods, the DLLME method introduced an automated liquid handling workstation to streamline the entire operational process, thereby eliminating potential manual errors. Notably, most current methods are manual and operate in a single-sample mode. Conversely, the DLLME method could simultaneously process four samples, thereby significantly enhancing experimental efficiency while maintaining rigorous error control. Regarding solvent usage, the DLLME method employed a blend of natural fatty acids and bio-based solvents. No toxic reagents such as acetone, H₂SO₄, and acetonitrile were used. The DLLME method necessitated only 183 μL of octanoic acid and 367 μL of GVL, resulting in a substantial reduction in solvent consumption and better alignment with green chemistry principles. Besides, extraction and phase separation were achieved within only 5 min, eliminating the need for energy-intensive steps and time-consuming processes such as ultrasonication, vortexing, and centrifugation, thereby further enhancing the experiment's environmental friendliness. During the detection stage, the DLLME method utilized UHPLC-MS/MS as the detection instrument. When compared to traditional HPLC-UV and HPLC-DAD, this method could rapidly detect ten TFs in food samples within 5.5 min with notably lower LOD and LOQ, offering superior detection speed and sensitivity. In summary, the DLLME method combines eco-friendliness, high sensitivity, and simplicity for TF detection in food matrices.

**Table 3:** Comparative analysis of DLLME versus published extraction methods of TFs in food samples in the last three years.

Sample preparation						Detection performance			Ref.
Extraction method	Automation level	Sample throughput	Sample	Solvent (μL)	Device (min)	Instrument	LOD (μg L^−1^)	LOQ (μg L^−1^)	
DSPE	Manual	1	Water	Acetone (2400)	Sonicator (5) Centrifuge (NG)	HPLC-UV	0.6–1.0	2.0–3.2	(Zhang, Tang, et al., 2023)
PGS-SUPRAME	Manual	1	Water Honey Milk	1-Dodecanol (100)	Centrifuge (3)	HPLC-UV	10–30	30–90	(Jaroensan et al., 2023)
VA-DLLME	Manual	1	Juice	HDES-FF (113.6)	Sonicator (20) Vortex mixer (1)	HPLC-DAD	250	800	(Na et al., 2023)
μ-SPE	Manual	1	Water Juice Milk Beverage	Sodium dodecyl sulphate (150)	Centrifuge (10) Vortex mixer (0.3)	HPLC-UV	3	9	(Kachangoon, Vichapong, et al., 2024)
EA-SDES-LPME	Manual	1	Water Beverage	SDES (400) H_2_SO_4_ (1200) Na_2_CO_3_ (2800)	Vortex mixer (1) Centrifuge (3)	HPLC- UV	1–2	5–10	(Zhang, Ren, et al., 2023)
SD-DLLME	Manual	1	Wine	Acetonitrile (1000) 1-Octanol (100)	Vortex mixer (1)	UHPLC-MS/MS	0.03	0.1	(Bernardi et al., 2022)
DLLME	Automated	4	Water Juice Wine Tea	Octanoic acid (183) γ-Valerolactone (367)	–	UHPLC-MS/MS	0.003	0.01	Current research

DSPE: Dispersive solid phase microextraction; PGS-SUPRAME: Popping candy-generated carbon dioxide and sugaring-out-assisted supramolecular solvent-based microextraction; VA-DLLME: Vortex-assisted dispersive liquid–liquid microextraction; μ-SPE: Micro solid phase extraction; EA-SDES-LPME: Effervescence-assisted switchable deep eutectic solvent-based liquid phase microextraction; SD-DLLME: Solvent demulsifcation-dispersive liquid-liquid microextraction.

## 4 Conclusion

An automated high-throughput DLLME method was developed based on an automated liquid handling workstation. Combined with UHPLC-MS/MS, it enabled efficient detection of TFs in food samples, including water, juice, wine, and tea. Environmentally friendly octanoic acid was served as the extractant, while bio-based solvent GVL was employed as the dispersant; both solvents align well with the principles of green analytical chemistry. During the sample pre-treatment, the extraction process is fast and completes in a few seconds. The automated working pipette station ensured precise positioning and accurate transfer of trace liquids, thereby significantly enhancing throughput and sample preparation efficiency. Rapid phase separation was achieved by introducing a saturated sodium chloride solution as the demulsifier, effectively replacing the traditional time-consuming centrifugation step. By employing UHPLC-MS/MS, the proposed method achieved rapid quantification of ten TFs within 5.5 min. The linear calibration ranged from 0.01 to 1 μg L^−1^ with an *R*^2^ value greater than 0.996, indicating a strong linear relationship between the analyte concentration and the signal response. The intra-day and inter-day RSDs ranged from 2.0 to 6.5 % and 2.1–7.5 % respectively, indicating good repeatability of the method. The method exhibited excellent sensitivity, achieving a LOD as low as 0.003 μg L^−1^ and a LOQ of 0.01 μg L^−1^. In summary, this environmentally friendly, simple, and efficient DLLME method enhances the green and efficient characteristics of sample pre-treatment and provides a robust solution for pesticide residue analysis in complex matrices.

## CRediT authorship contribution statement

**Yuxin Wang:** Writing – original draft, Methodology, Investigation. **Jin Liu:** Data curation. **Suzhen Li:** Investigation. **Jizhen Fu:** Investigation. **Xiaowen Wang:** Supervision. **Li Li:** Validation, Project administration. **Xu Jing:** Writing – review & editing, Conceptualization.

## Informed consent

Not applicable.

## Declaration of competing interest

The authors declare no known competing financial interests or personal relationships that could have appeared to influence the work reported in this paper.

## Data Availability

Data will be made available on request.
