# Supplementary material for: Automated high-throughput dispersive liquid-liquid microextraction coupled with UHPLC-MS/MS for detecting triazole fungicides in water, juices, wine, and tea
Source: Food Chem X. 2025 Jun 30;29:102712. doi: 10.1016/j.fochx.2025.102712 (PMC12270822; doi:10.1016/j.fochx.2025.102712)
Supplement: Supplementary material [file mmc1.docx]

Supplementary material:

**Automated high-throughput dispersive liquid-liquid microextraction coupled with UHPLC-MS/MS for detecting triazole fungicides in water, juices, wine, and tea**

Yuxin Wang^a,b^, Jin Liu^a,b^, Suzhen Li^b^, Jizhen Fu^b^, Xiaowen Wang^a^, Li Li^b^, Xu Jing**^a,*^**

*^a^* *College of Food Science and Engineering,* *Shanxi Agricultural University, Taiyuan, Shanxi 030031, China;*

*^b^* *Shanxi Key Laboratory of Integrated Pest Management in Agriculture, College of Plant Protection, Shanxi Agricultural University, Taiyuan, Shanxi 030031, China;*

*Corresponding authors:

Li Li, E-mail: Shanxi Key Laboratory of Integrated Pest Management in Agriculture, College of Plant Protection, Shanxi Agricultural University, Shanxi 030031, China; E-mail: [sxaulili@sxau.edu.cn](mailto:sxaulili@sxau.edu.cn).

Xu Jing, College of Food Science and Engineering, Shanxi Agricultural University, Shanxi 030031, China; E-mail: [x.jing@vip.163.com](mailto:x.jing@vip.163.com).

Number of pages: 12

Number of figures: 6

Number of tables: 5


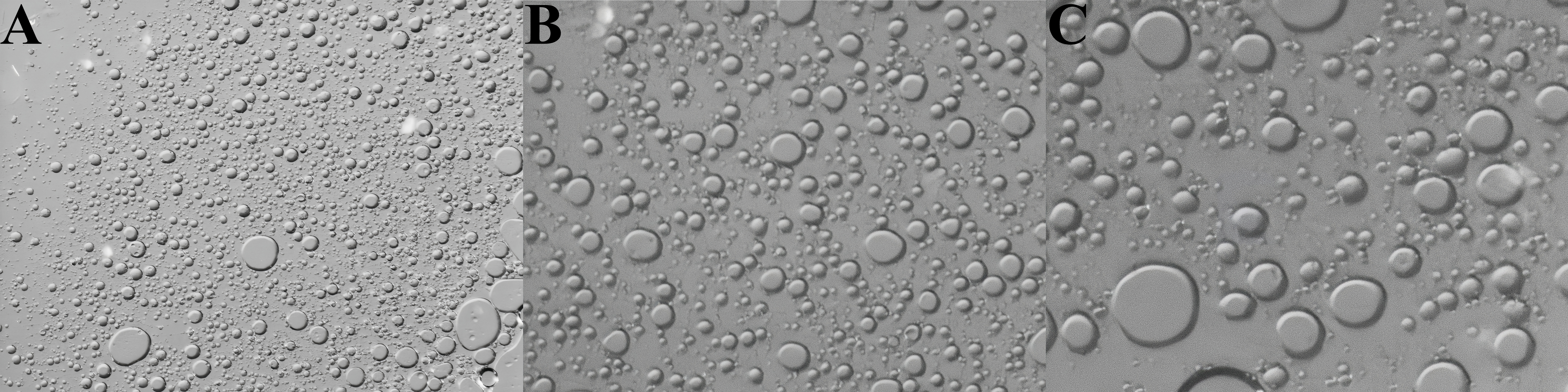


Fig. S1. Stereo microscope images at 40 X (A); 80 X (B); 135 X (C) magnification of the diluted emulsion.


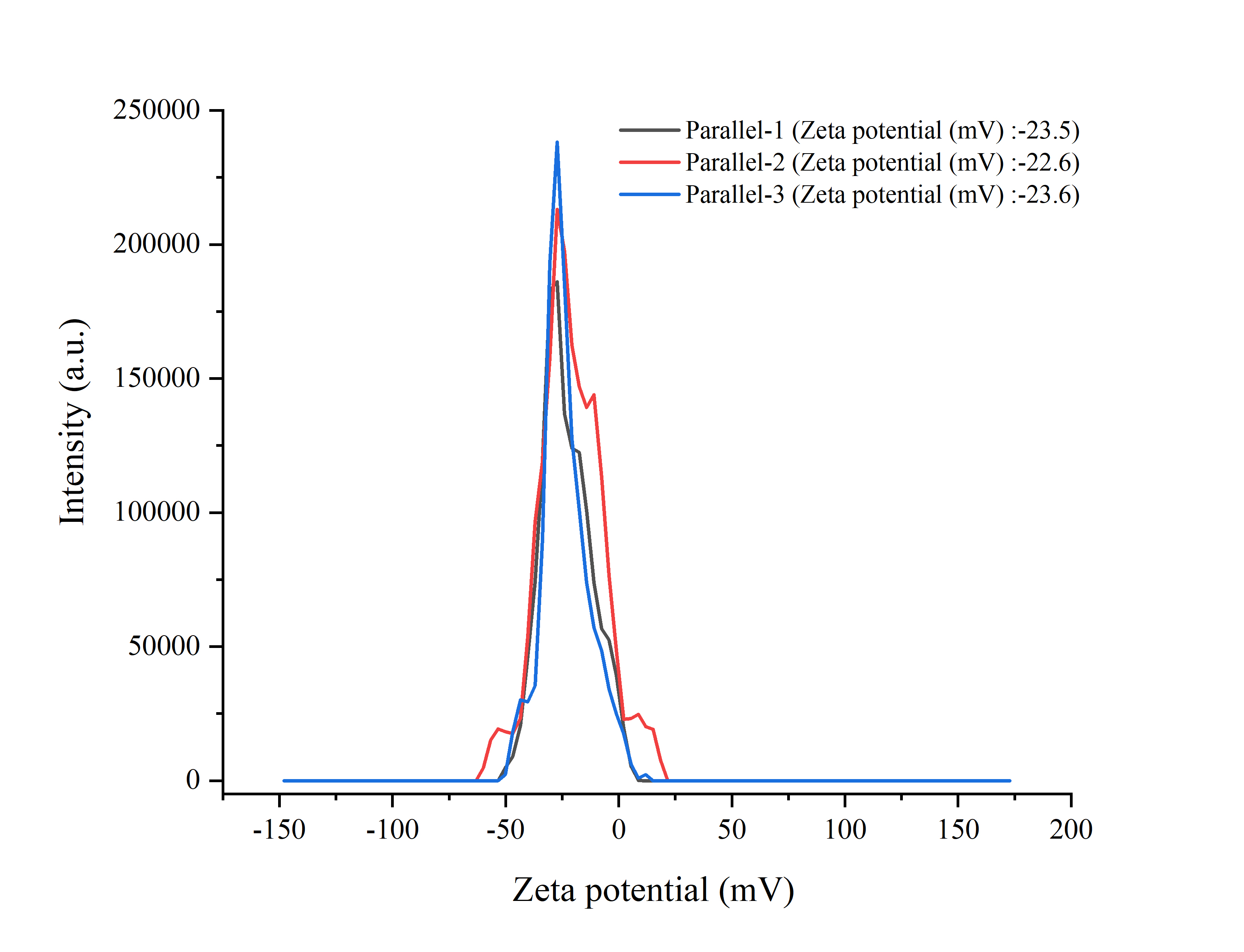


Fig. S2. Three parallel zeta potential plots of diluted emulsion.

**A**

**
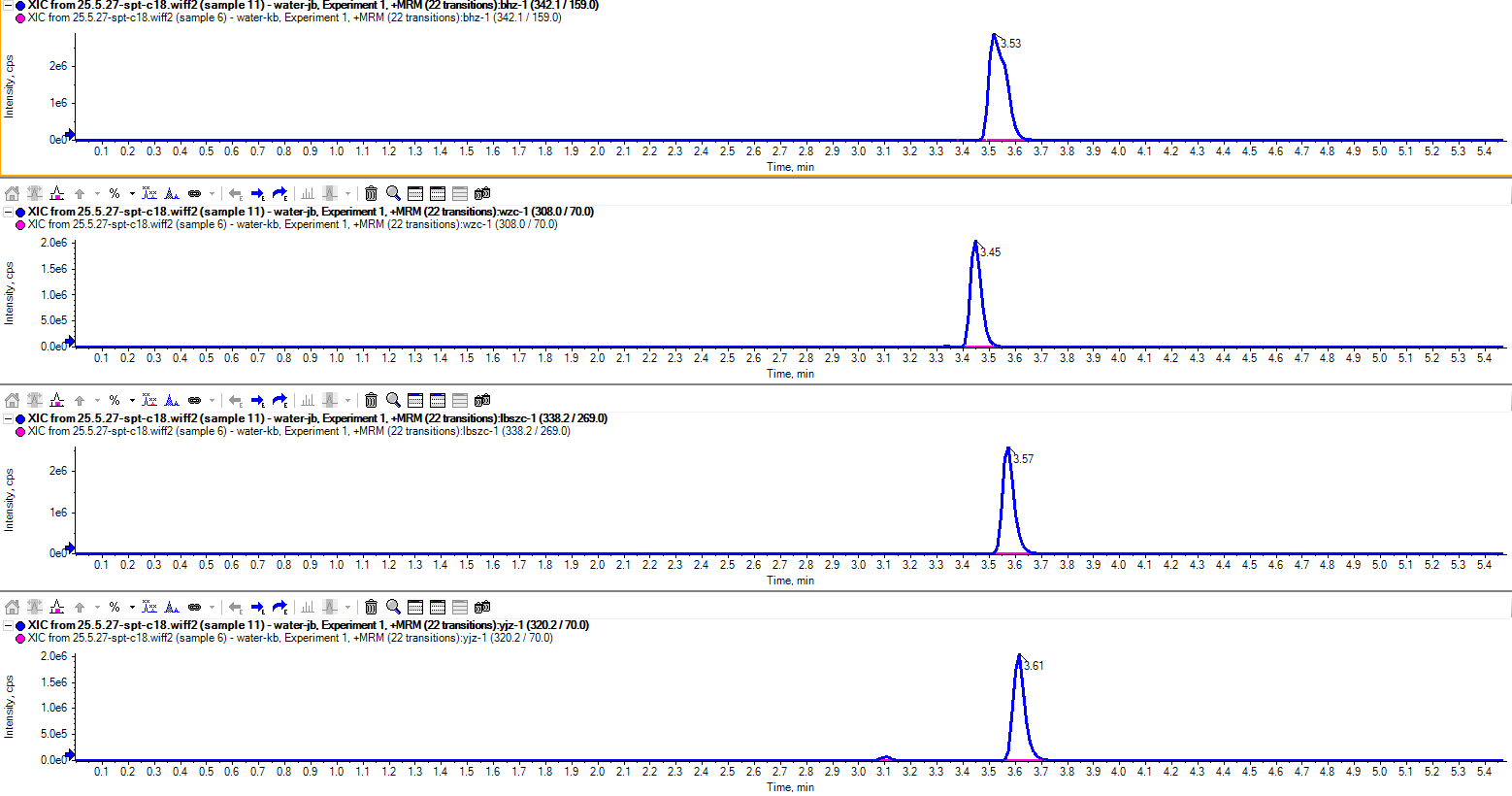
B**

**
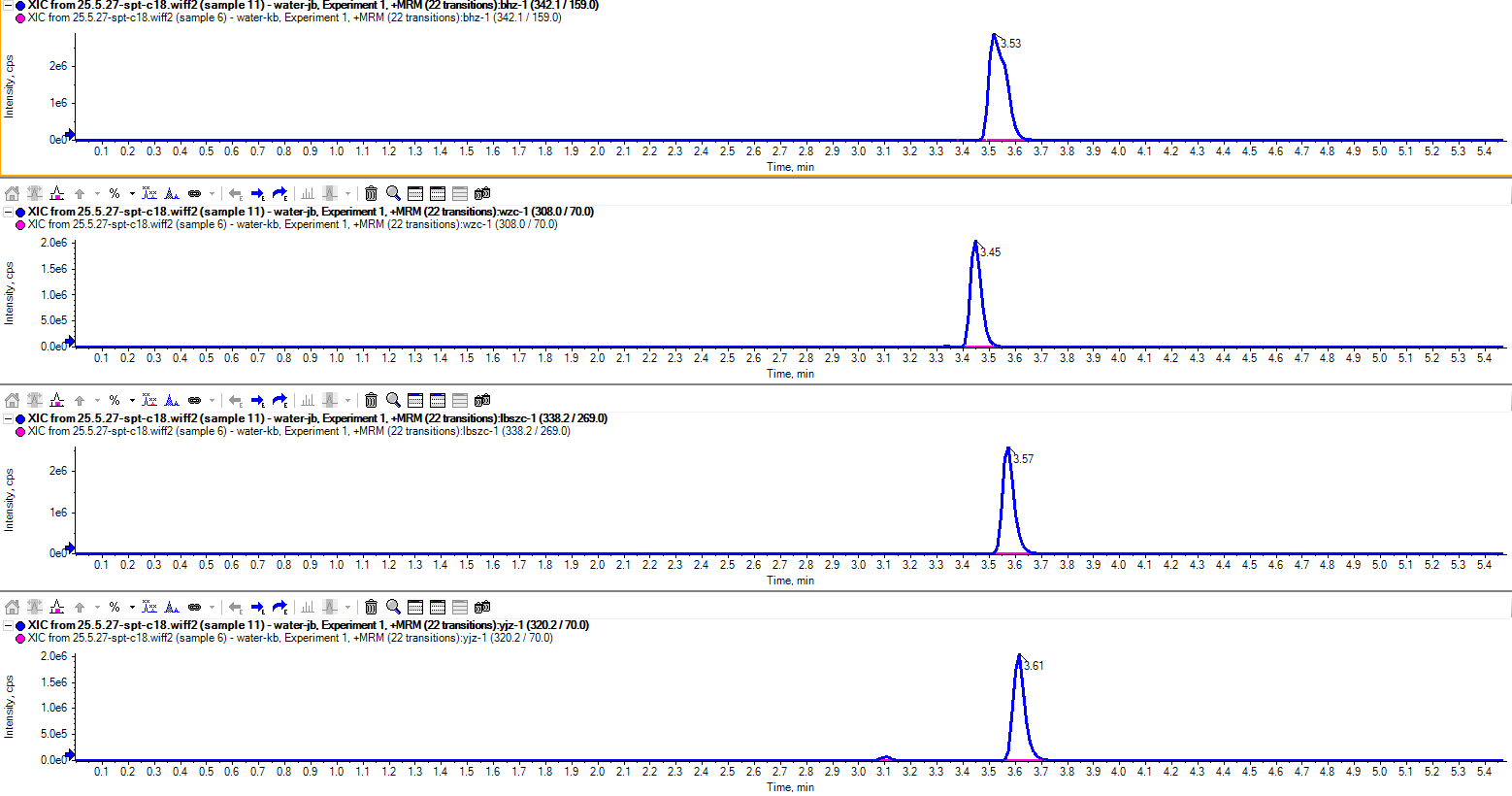
**

**C**

**
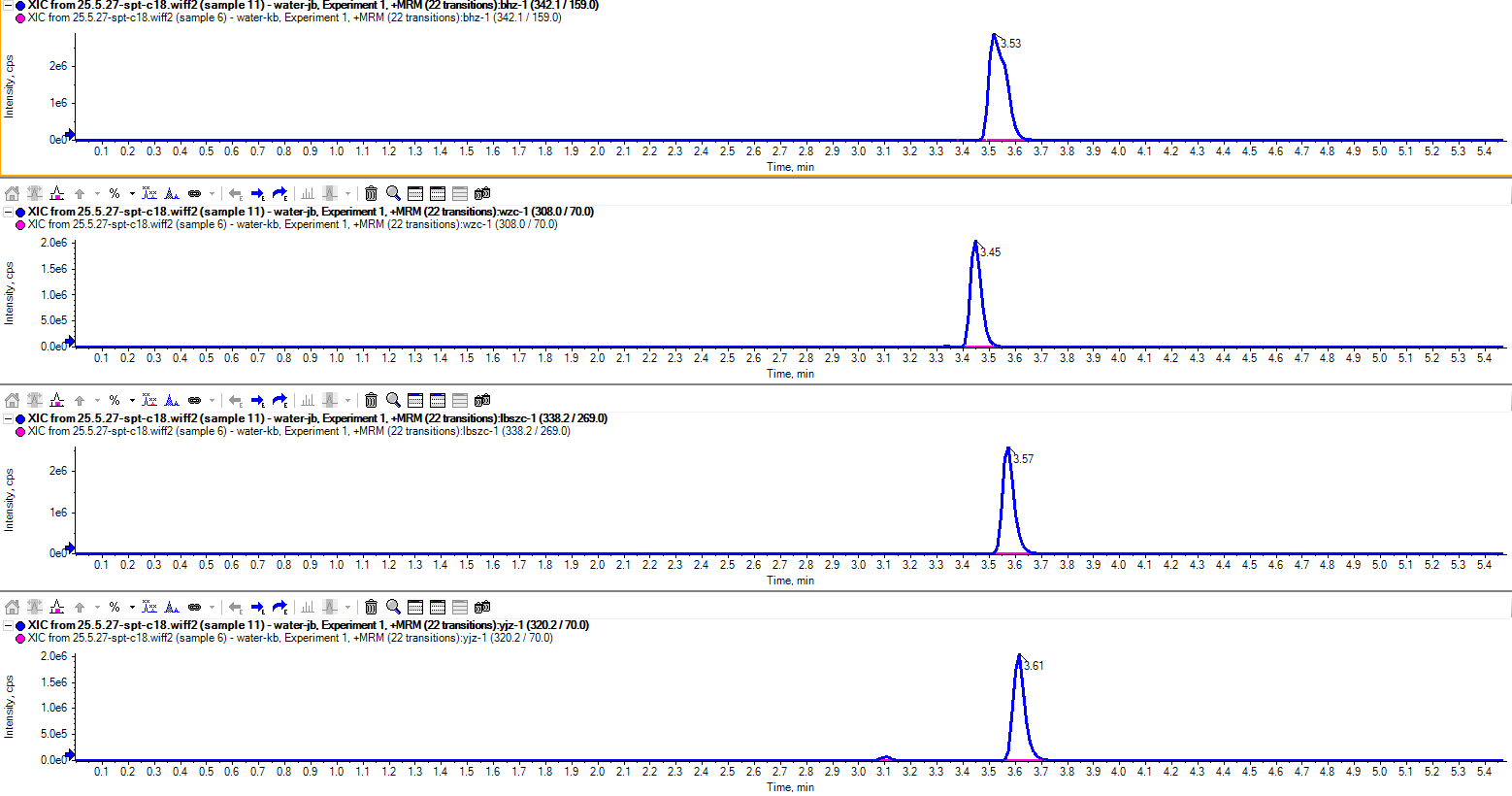
**

**D**

**
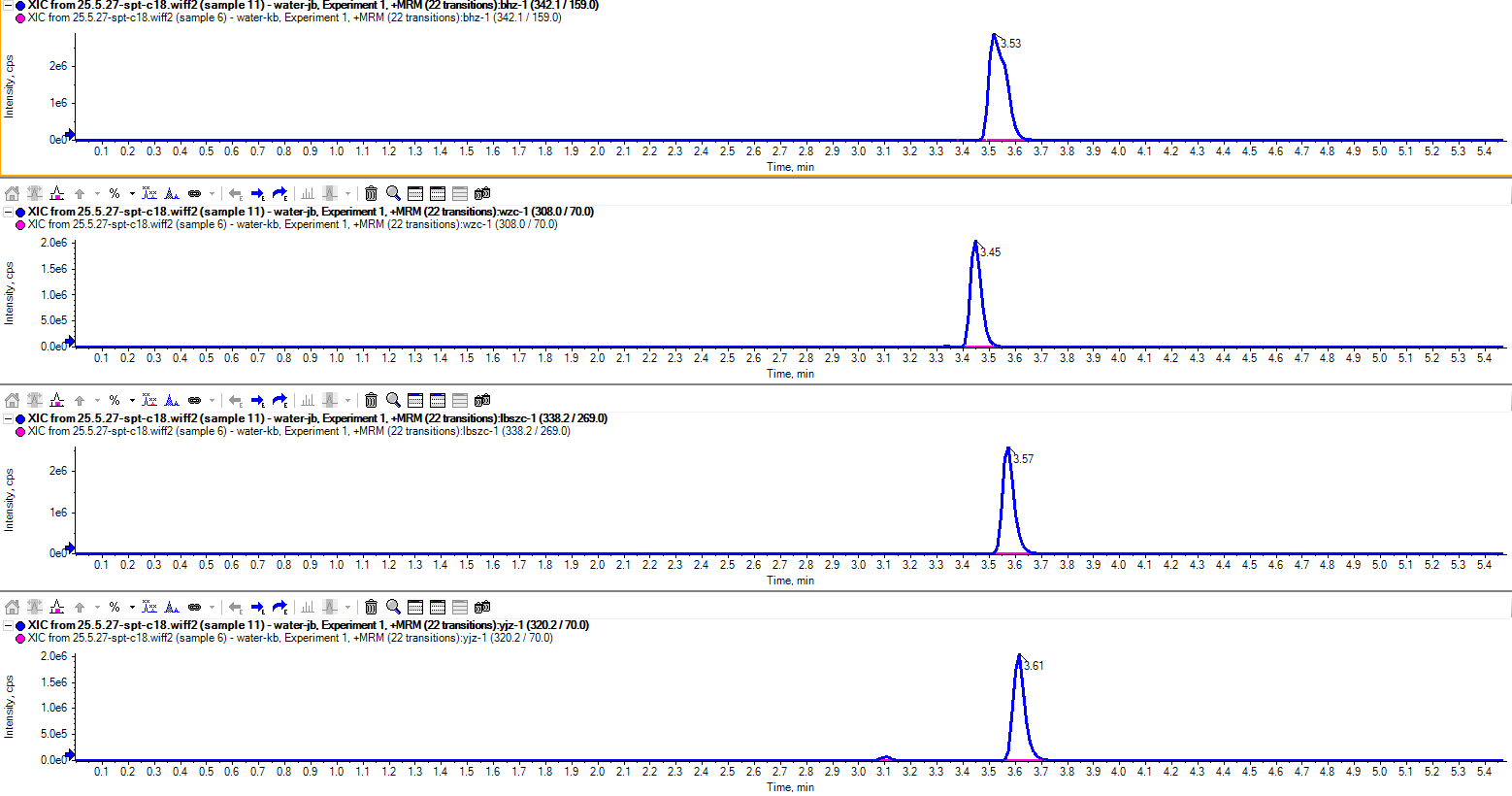
**

**E**

**
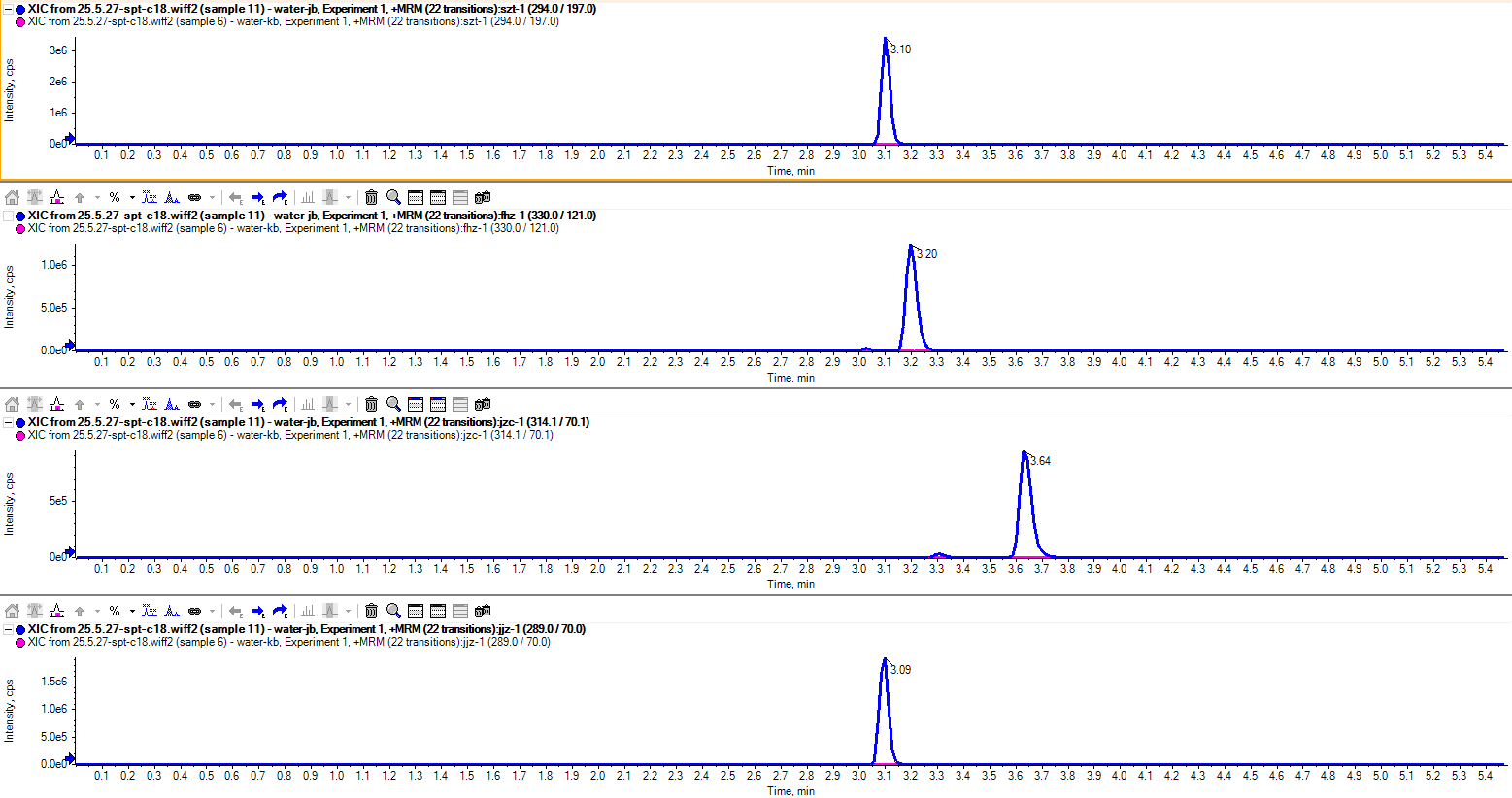
**

**F**

**
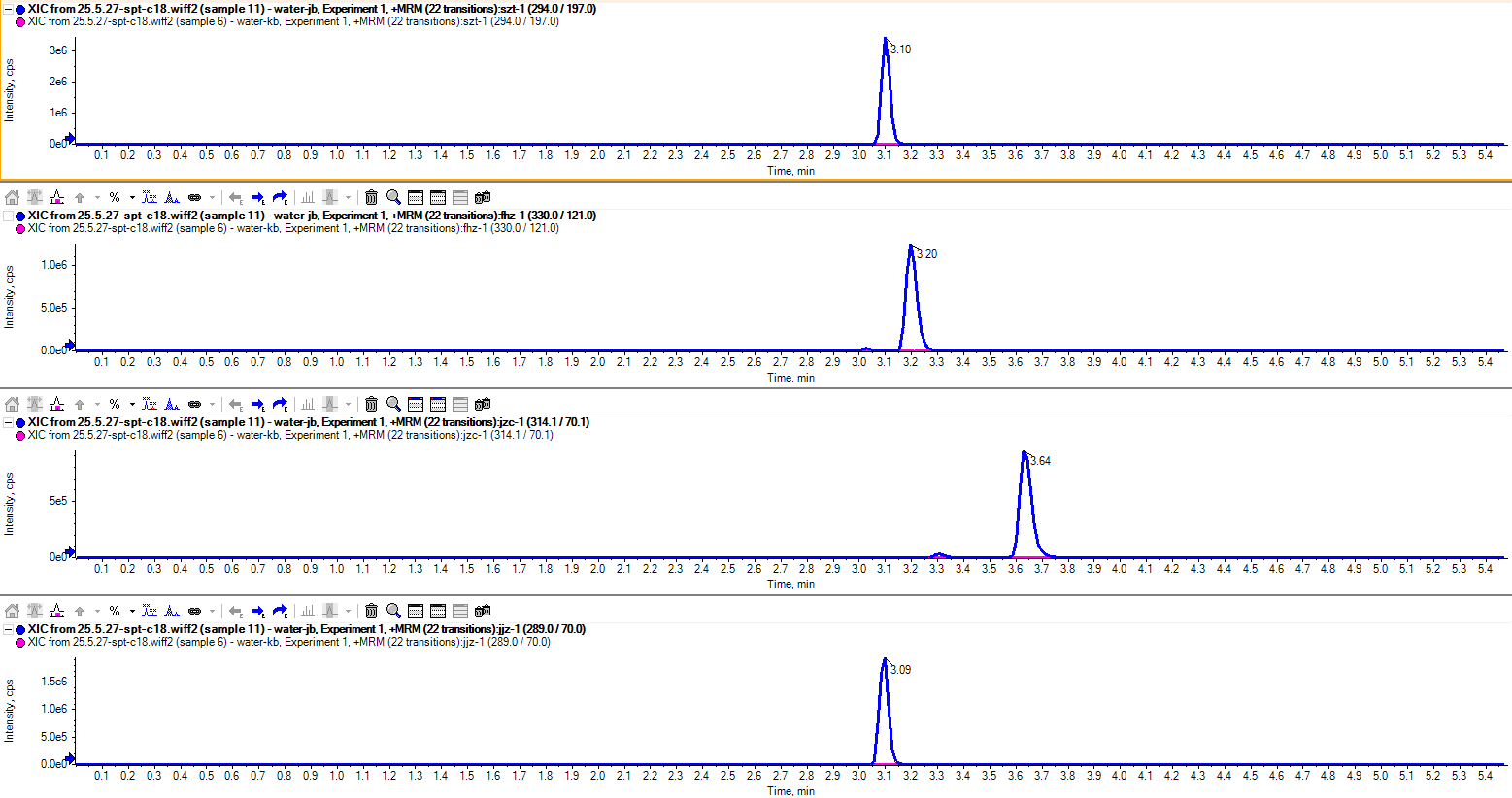
**

**G**

**
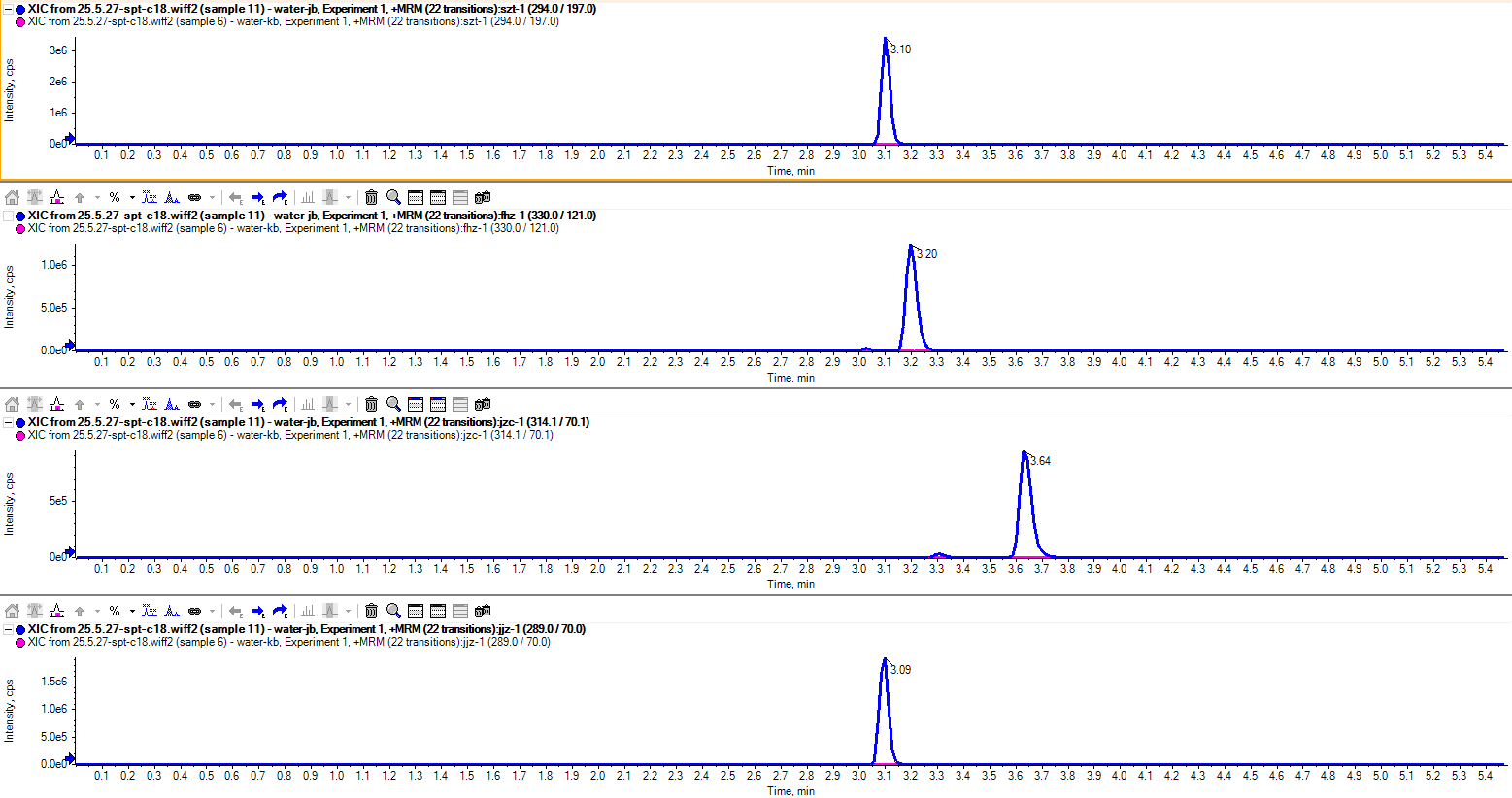
**

**H**

**
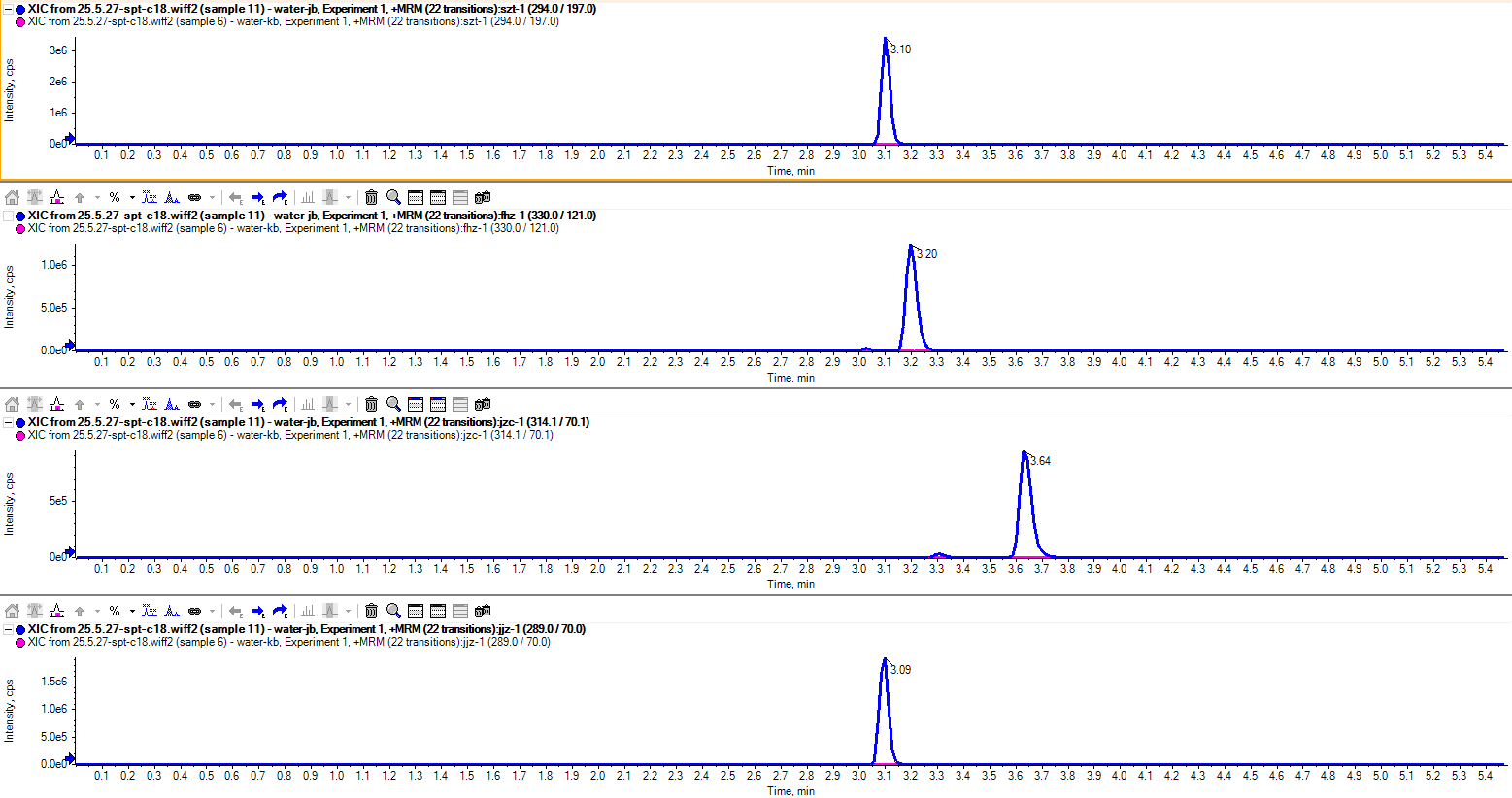
**

**I**

**
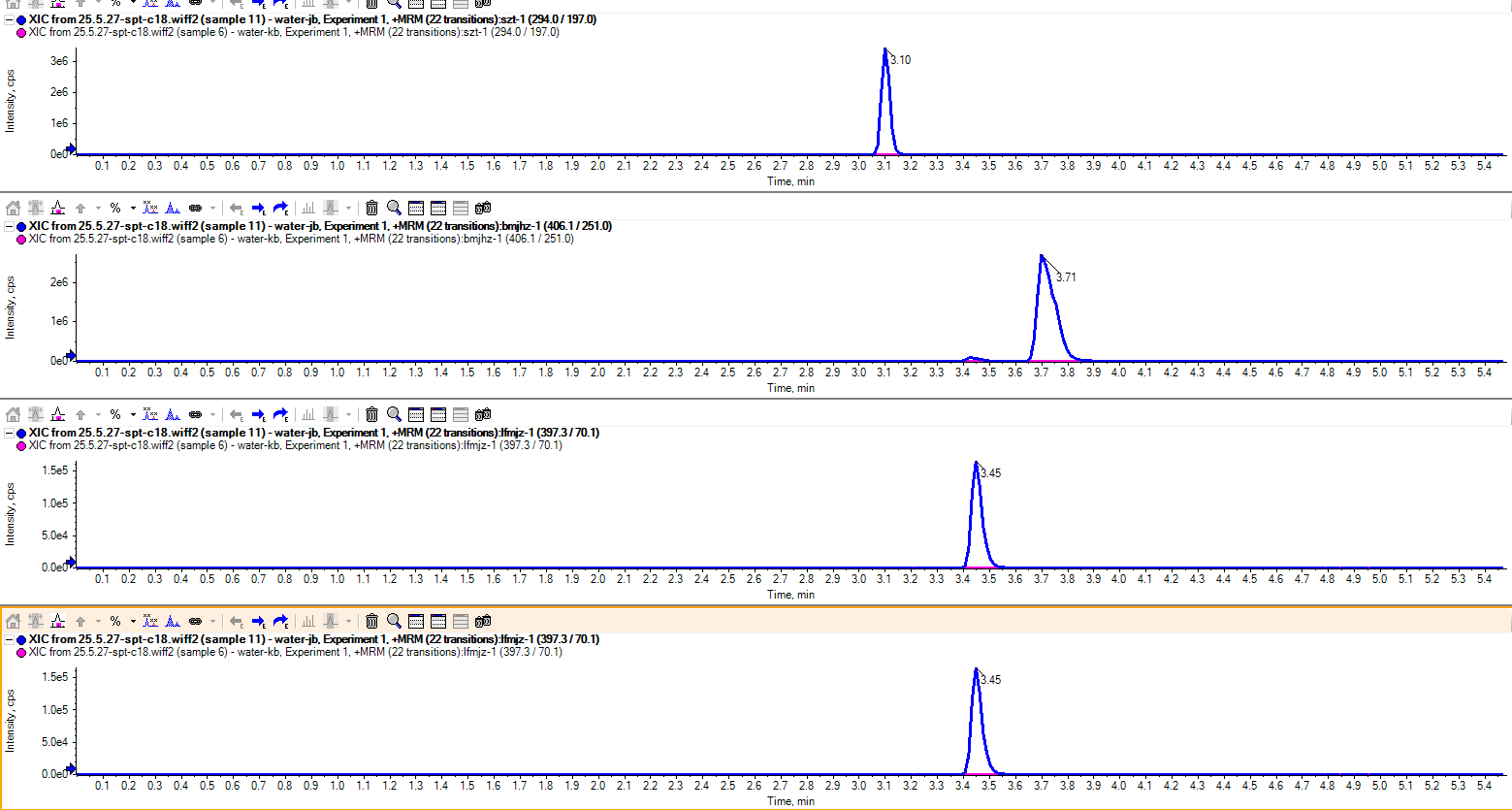
**

**J**

**
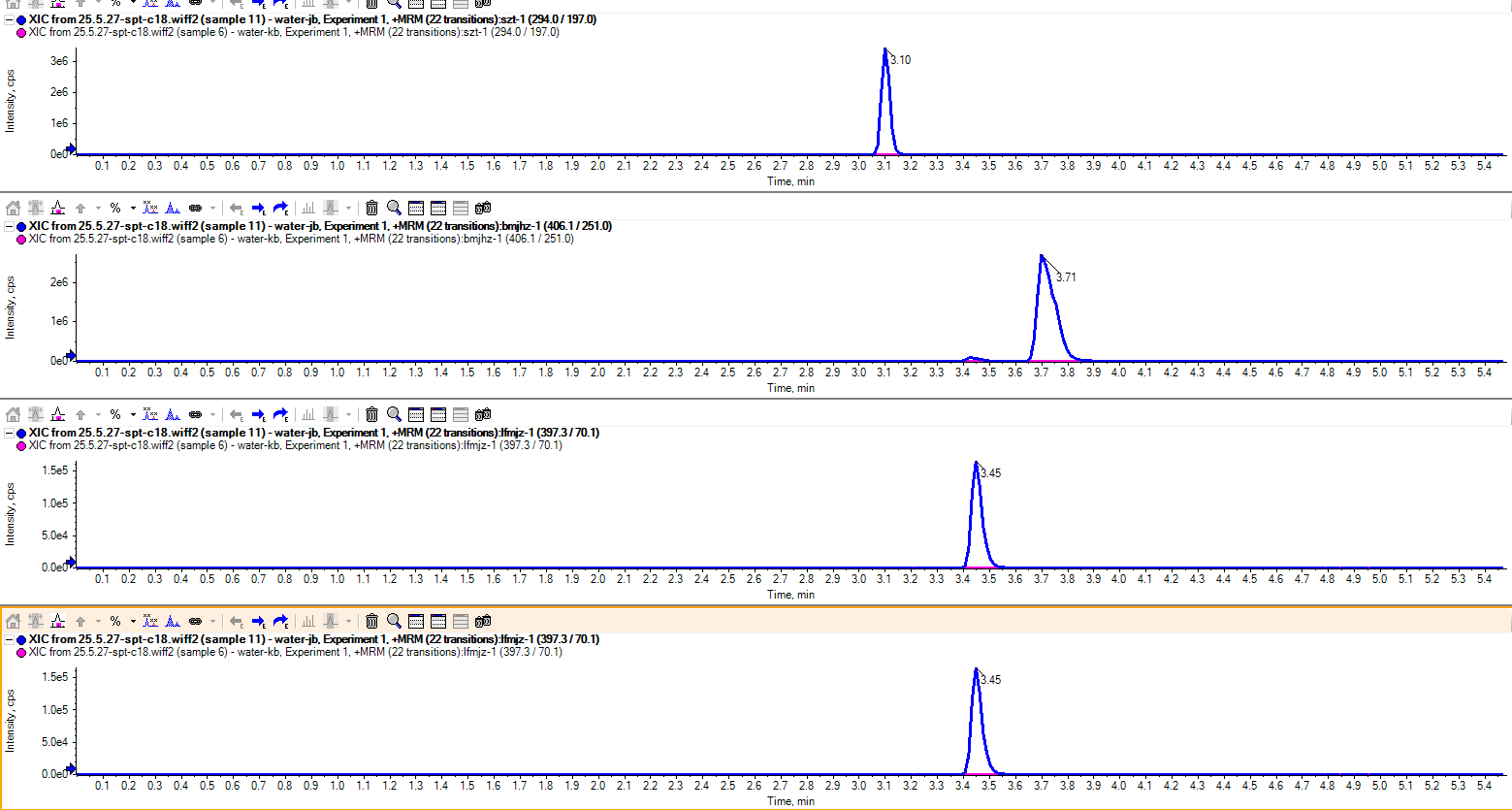
Fig. S3.** UHPLC-MS/MS chromatograms of ten TFs in water sample; the blue chromatograms represent the spiked water, while the pink chromatograms represent the blank water (A: Propiconazole; B: Tebuconazole; C: Bitertanol; D: Metconazole; E: Triadimefon; F: Epoxiconazole; G: Hexaconazole; H: Myclobutanil; I: Difenoconazole; J: Mefentrifluconazole).

**A**
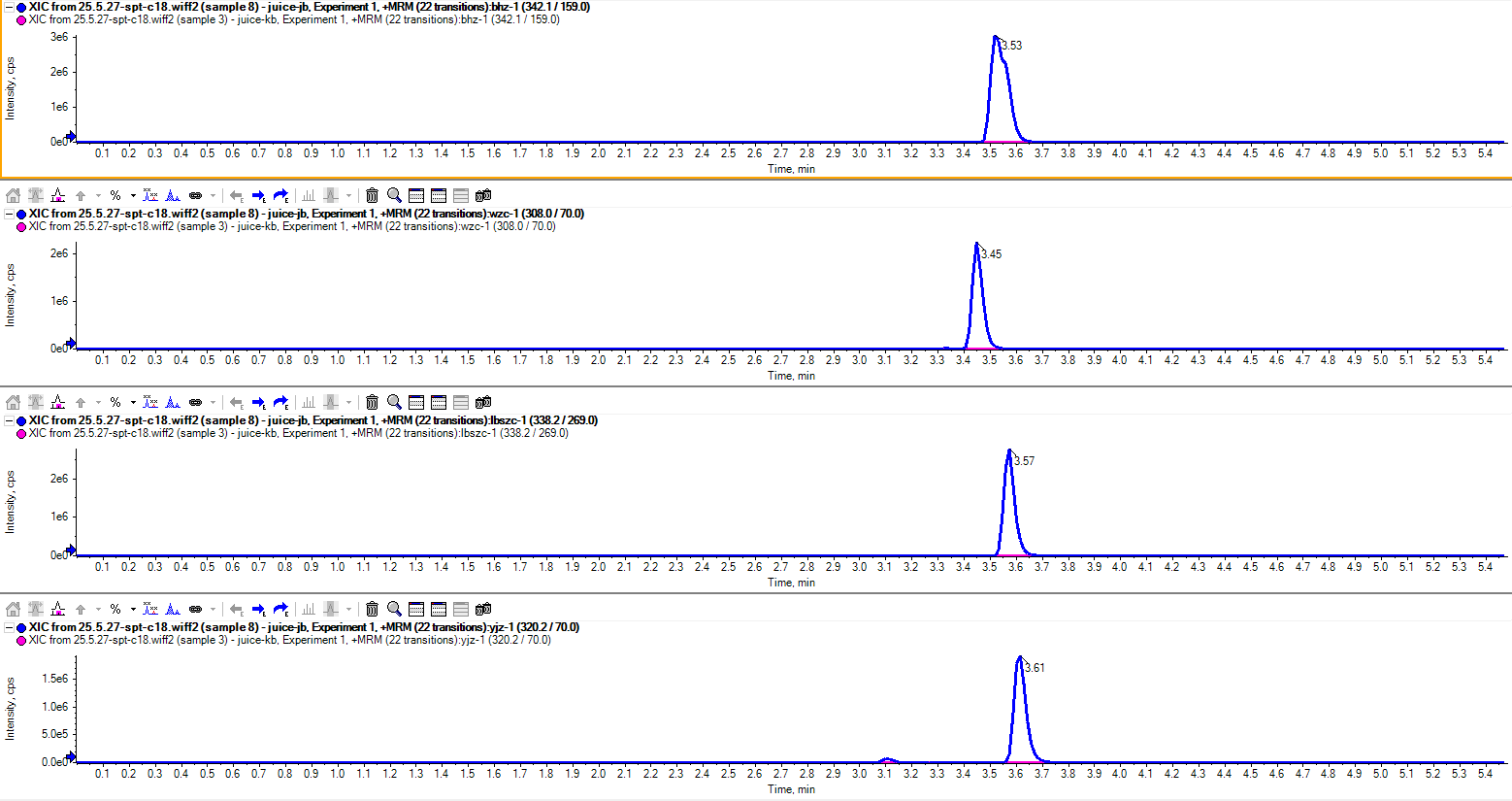


**B**
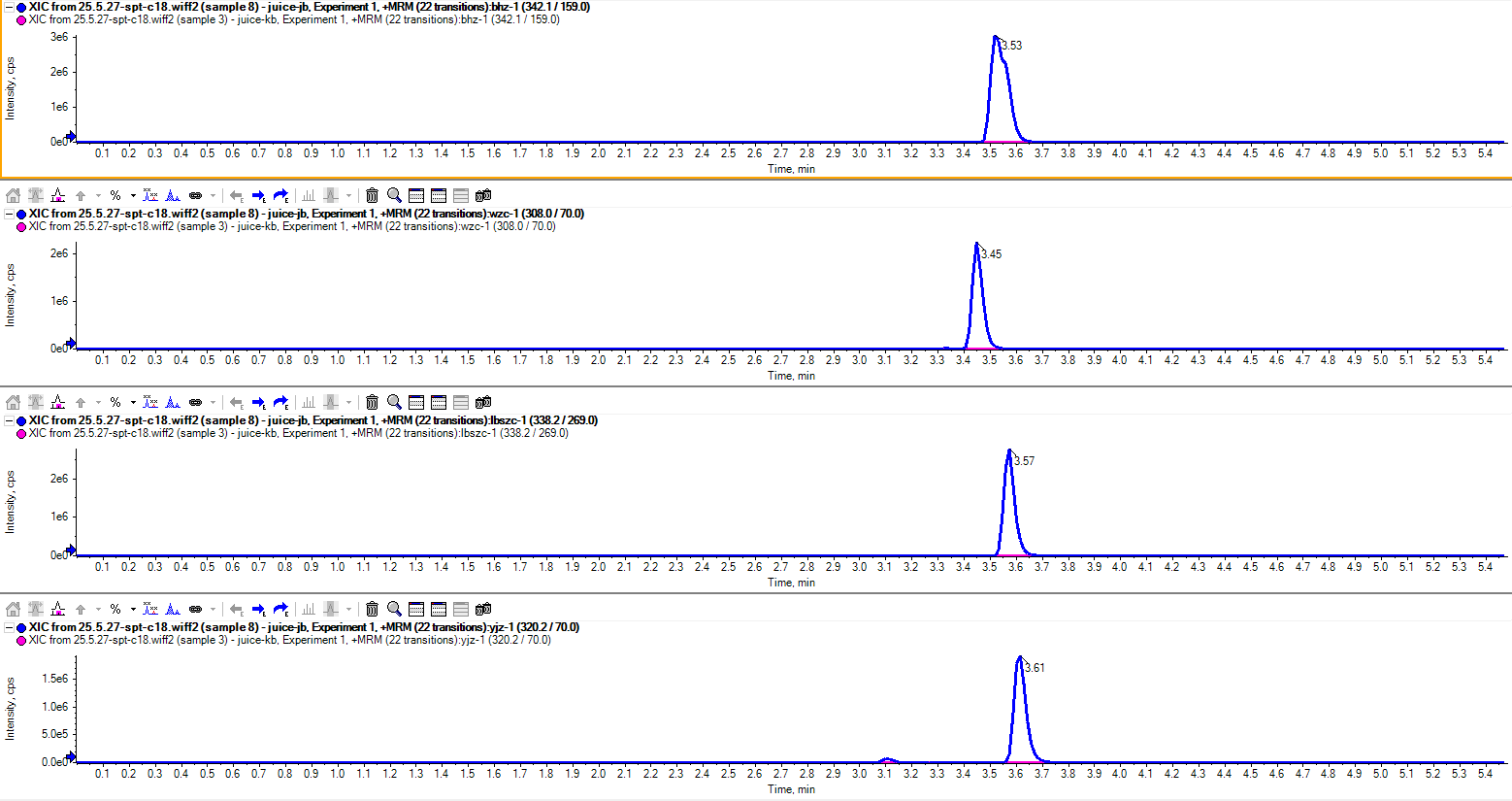


**C**


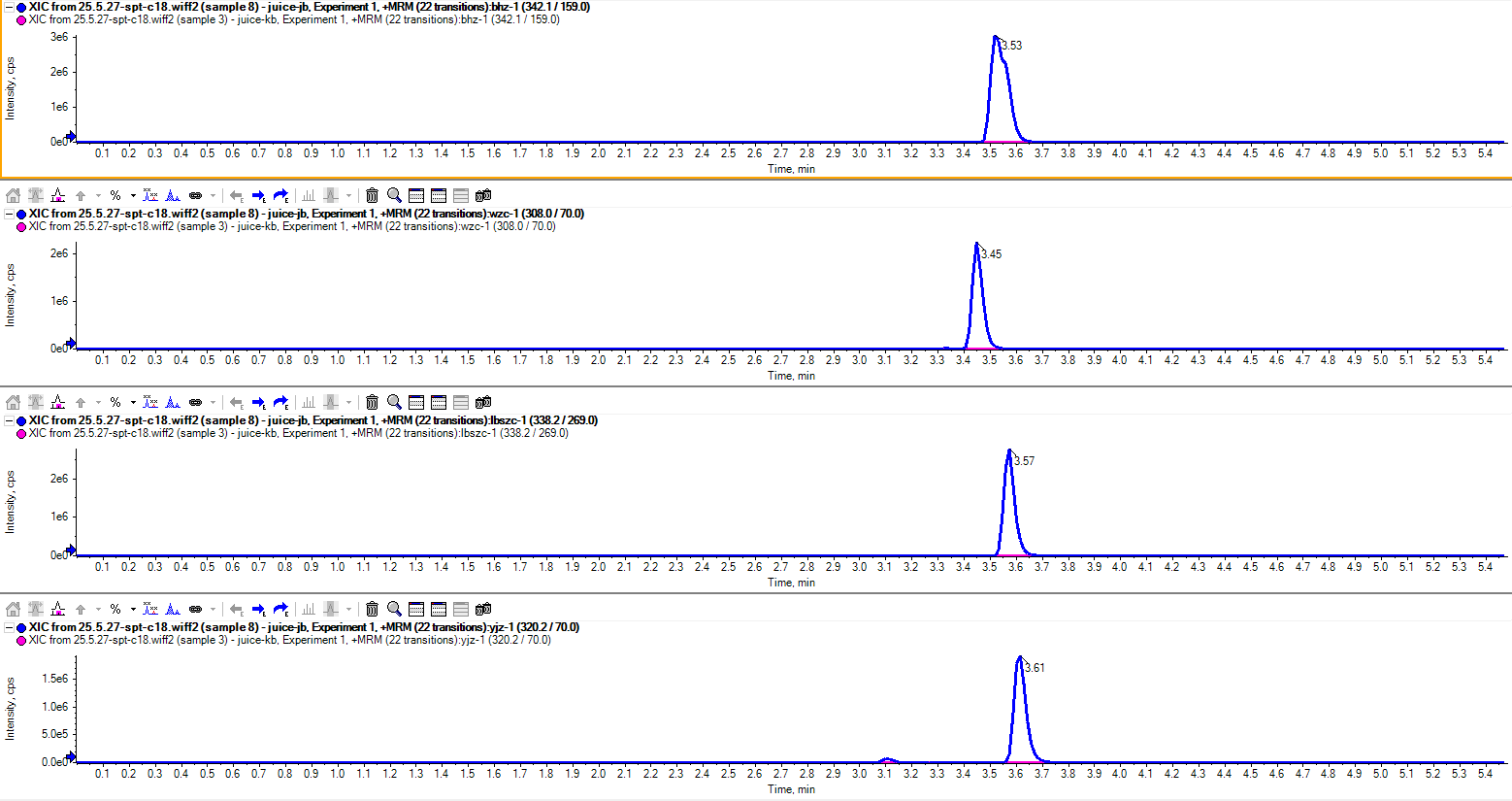


**D**


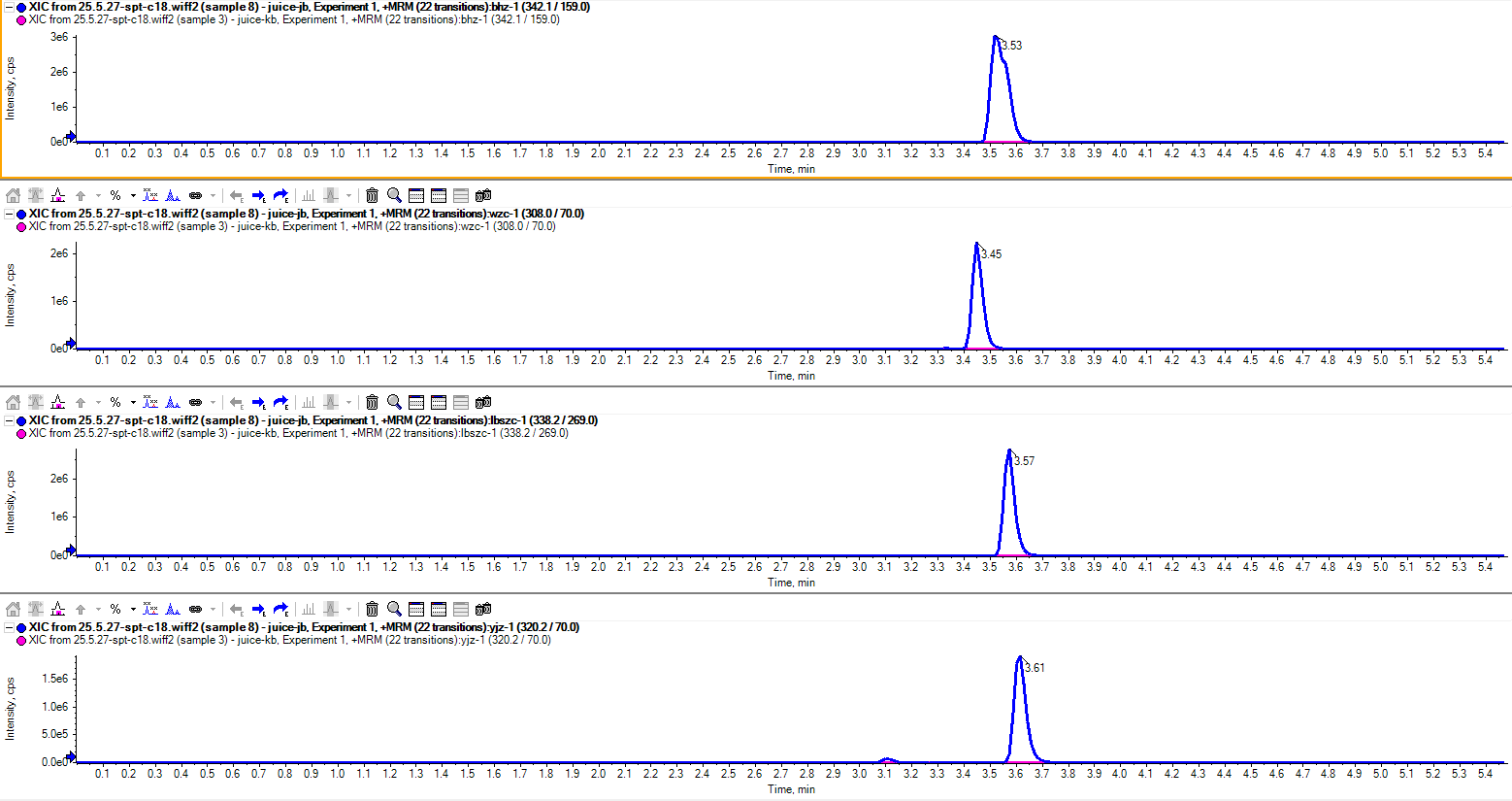


**E**


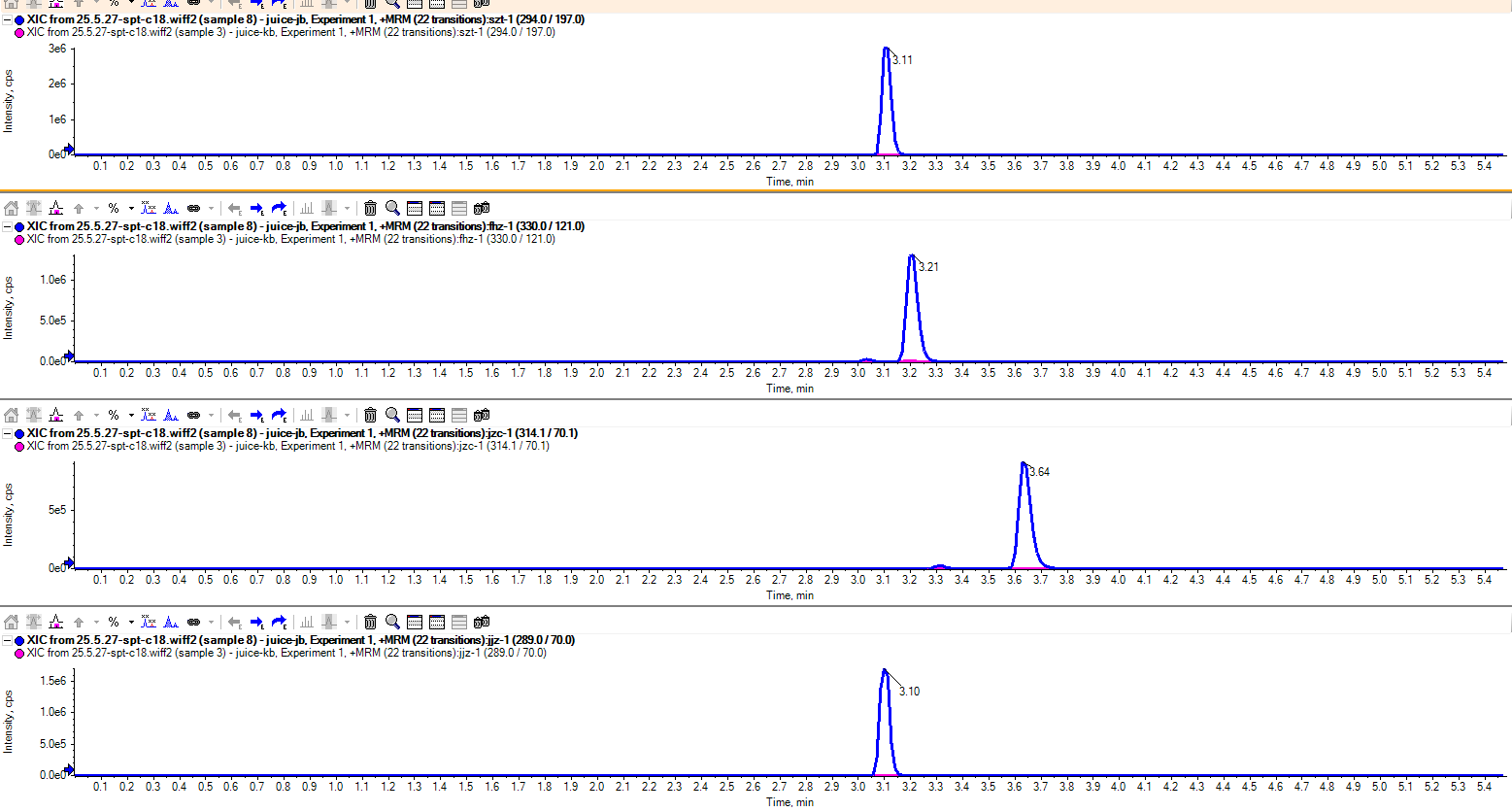


**F**


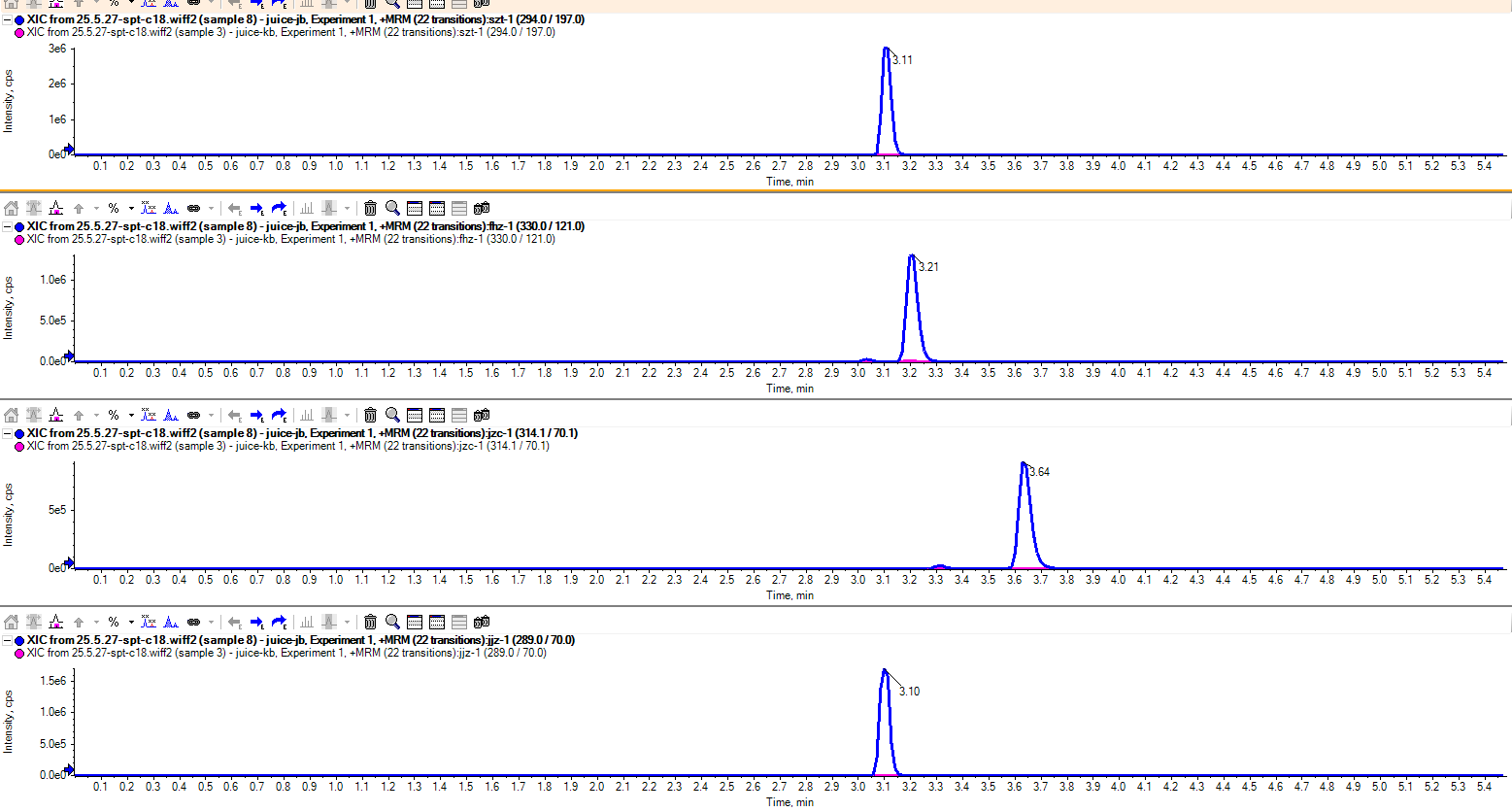


**G**


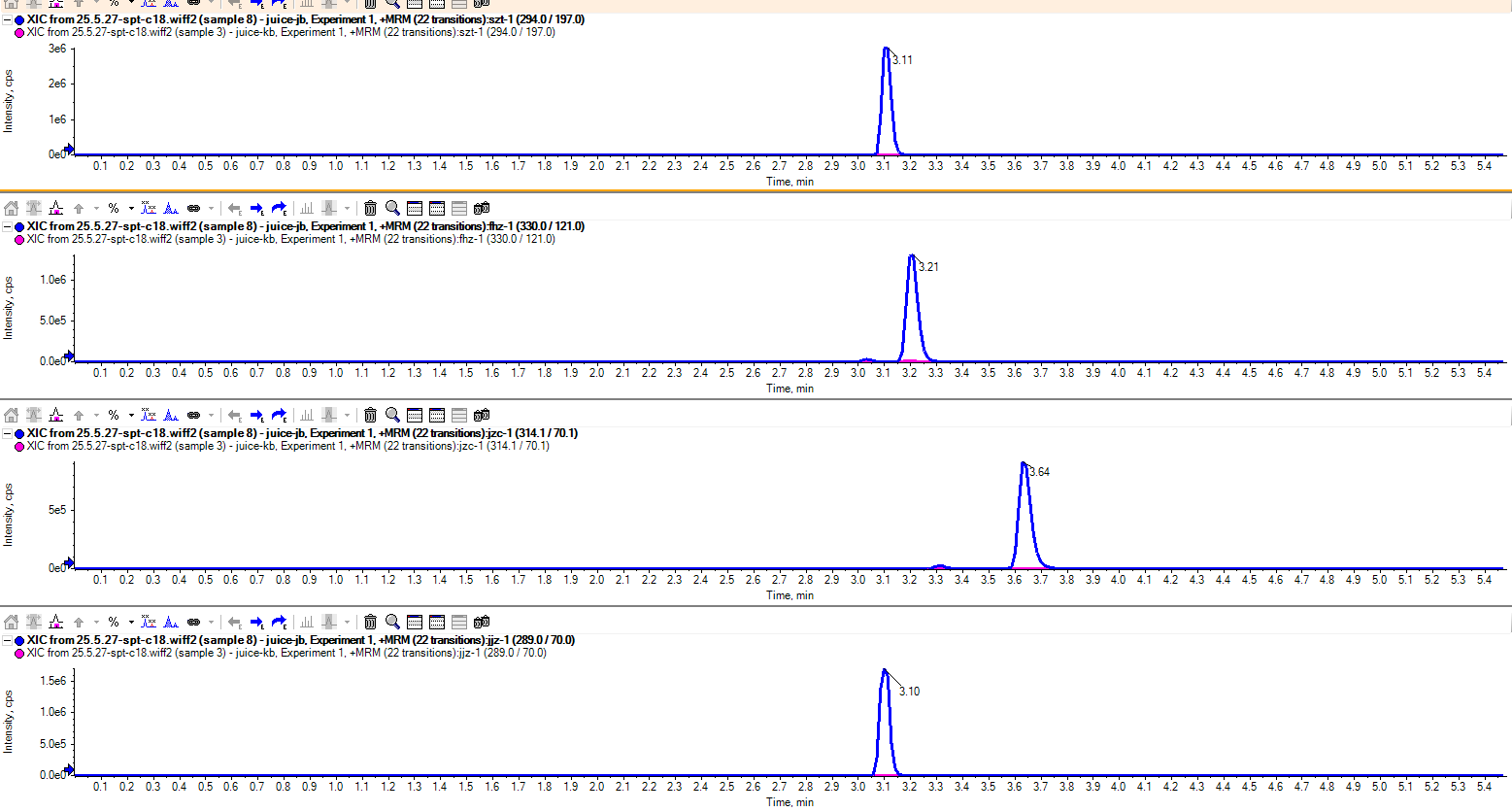


**H**
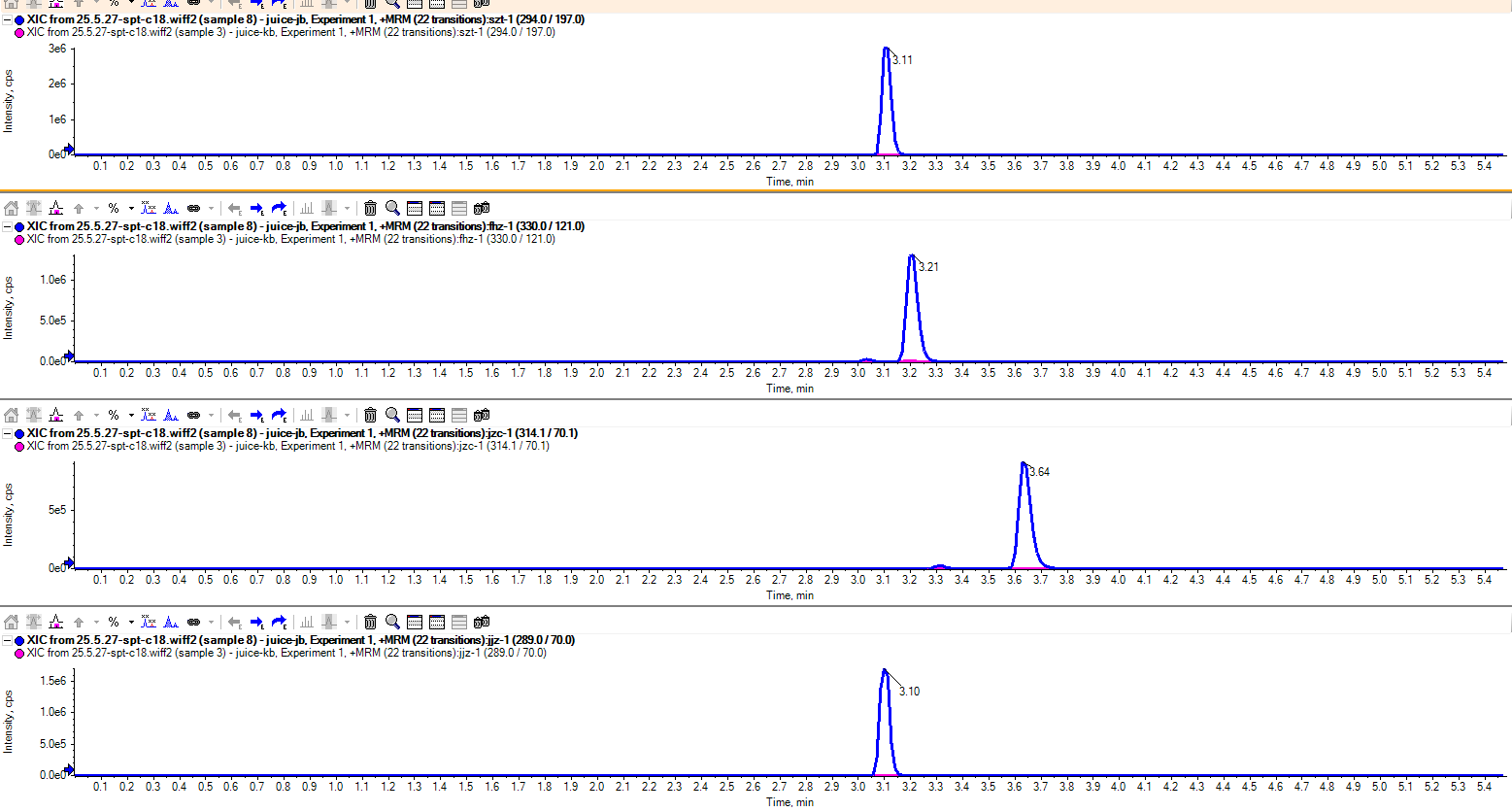


**I**
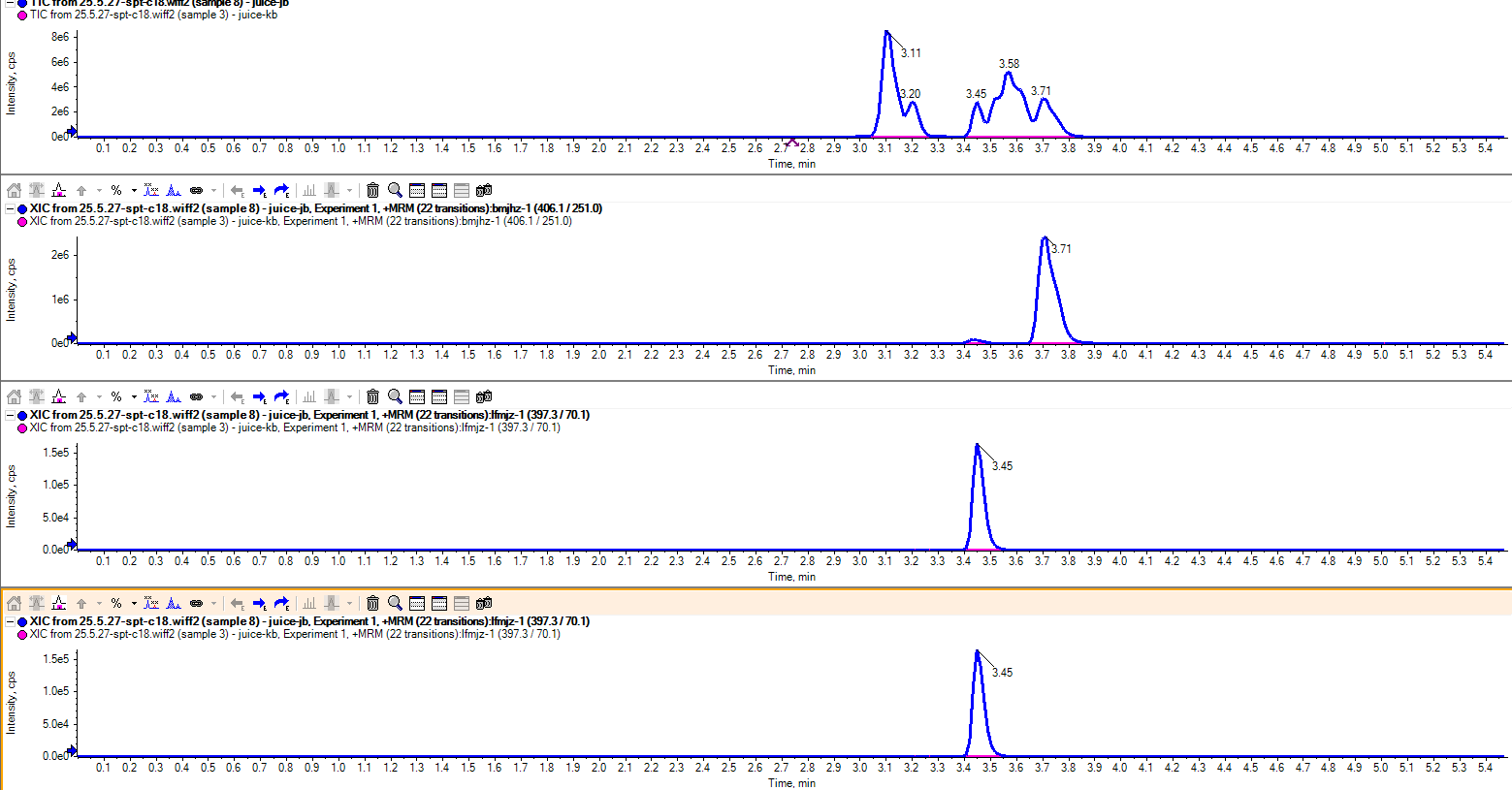


**J**


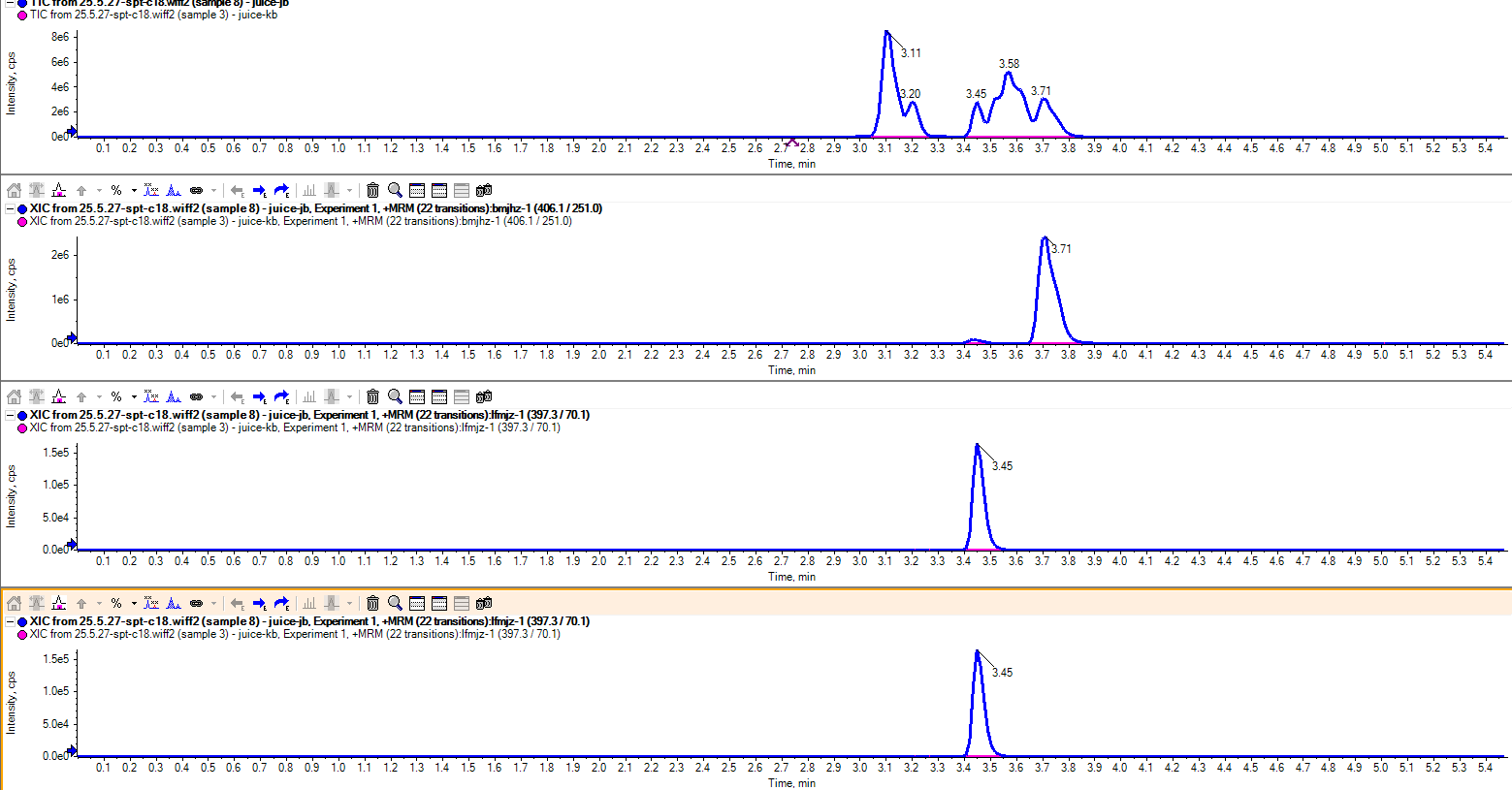


**Fig. S4.** UHPLC-MS/MS chromatograms of ten TFs in juice sample; the blue chromatograms represent the spiked juice, while the pink chromatograms represent the blank juice (A: Propiconazole; B: Tebuconazole; C: Bitertanol; D: Metconazole; E: Triadimefon; F: Epoxiconazole; G: Hexaconazole; H: Myclobutanil; I: Difenoconazole; J: Mefentrifluconazole).

**A**


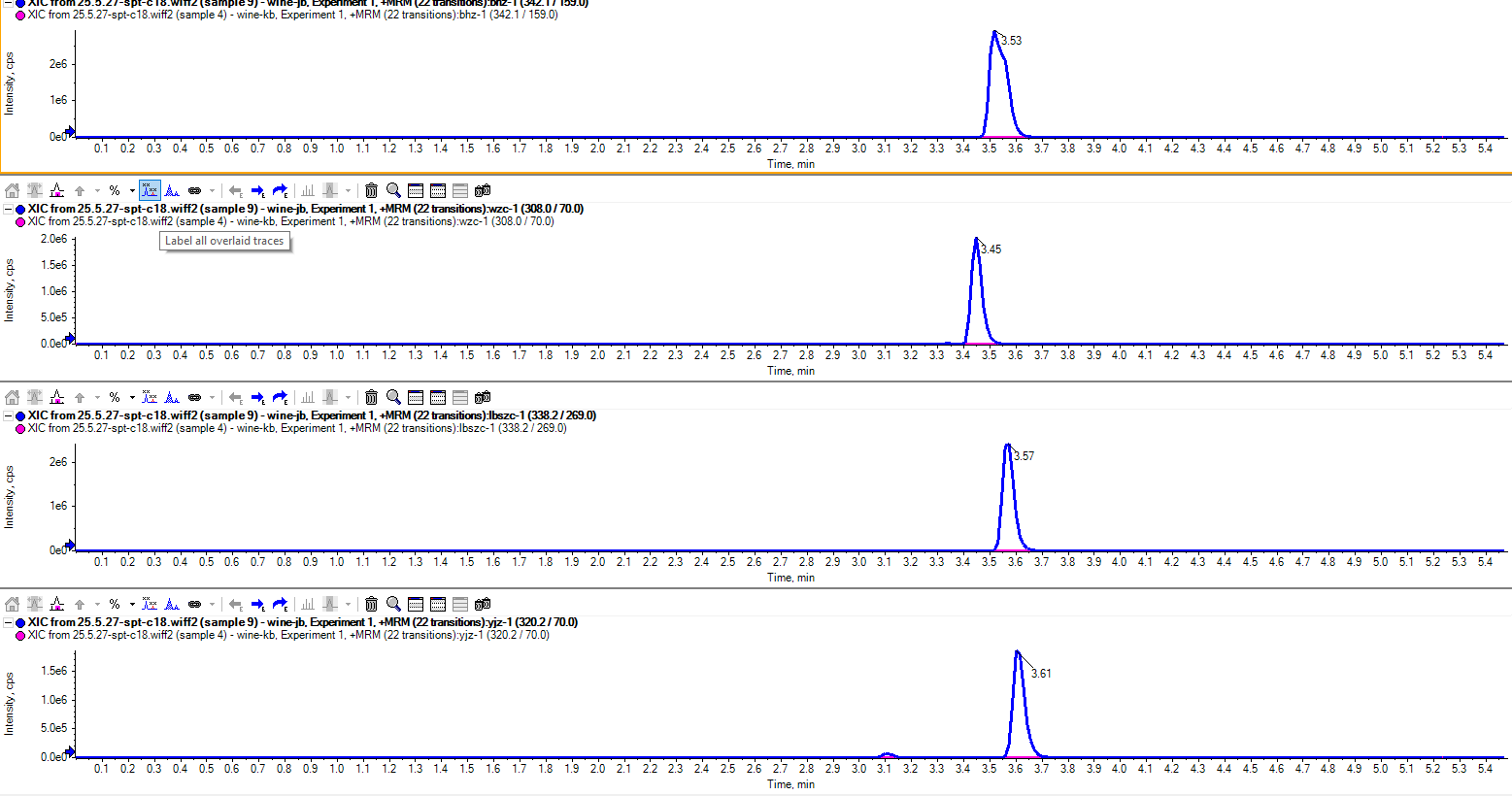


**B**


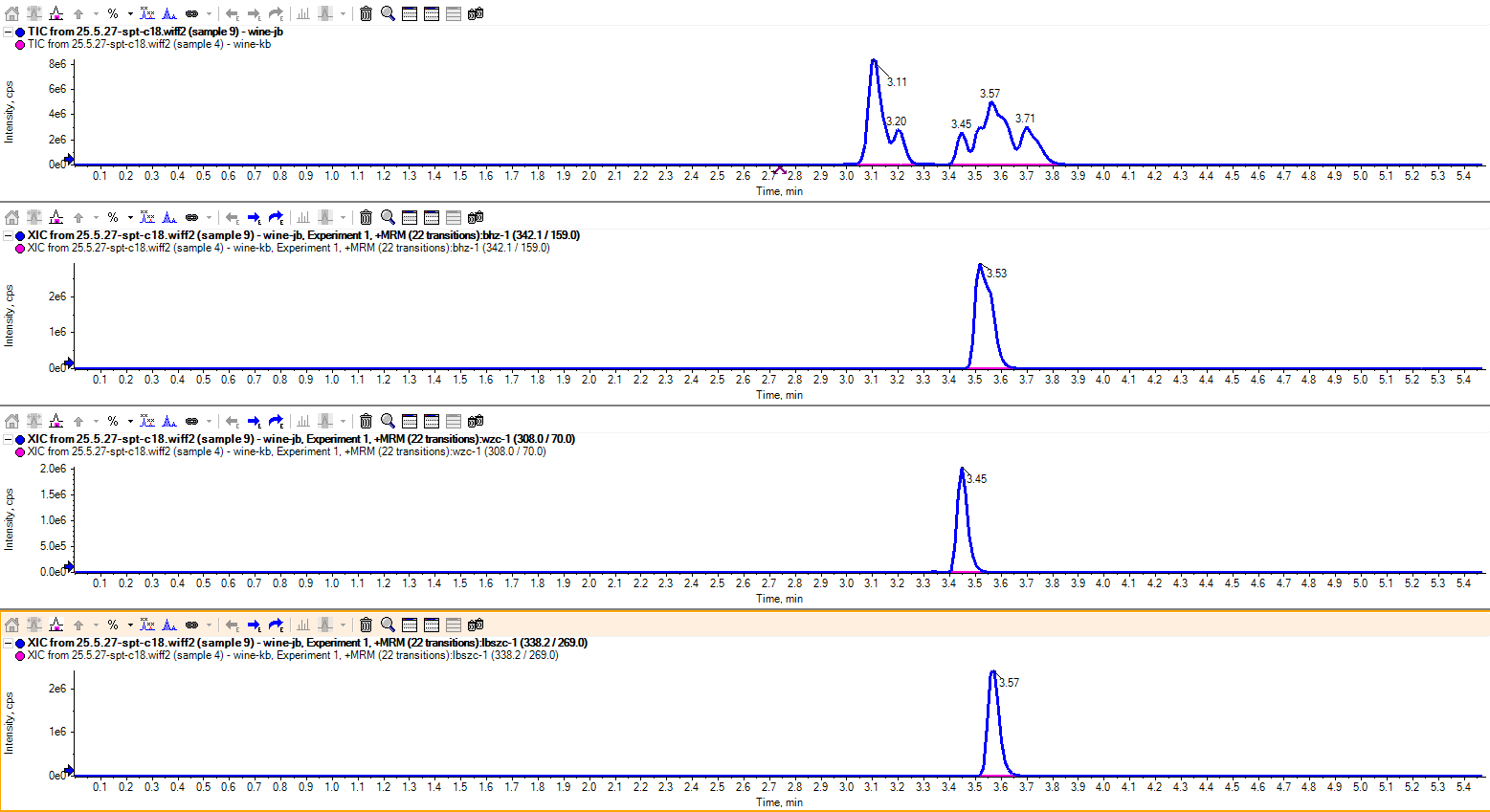


**C**


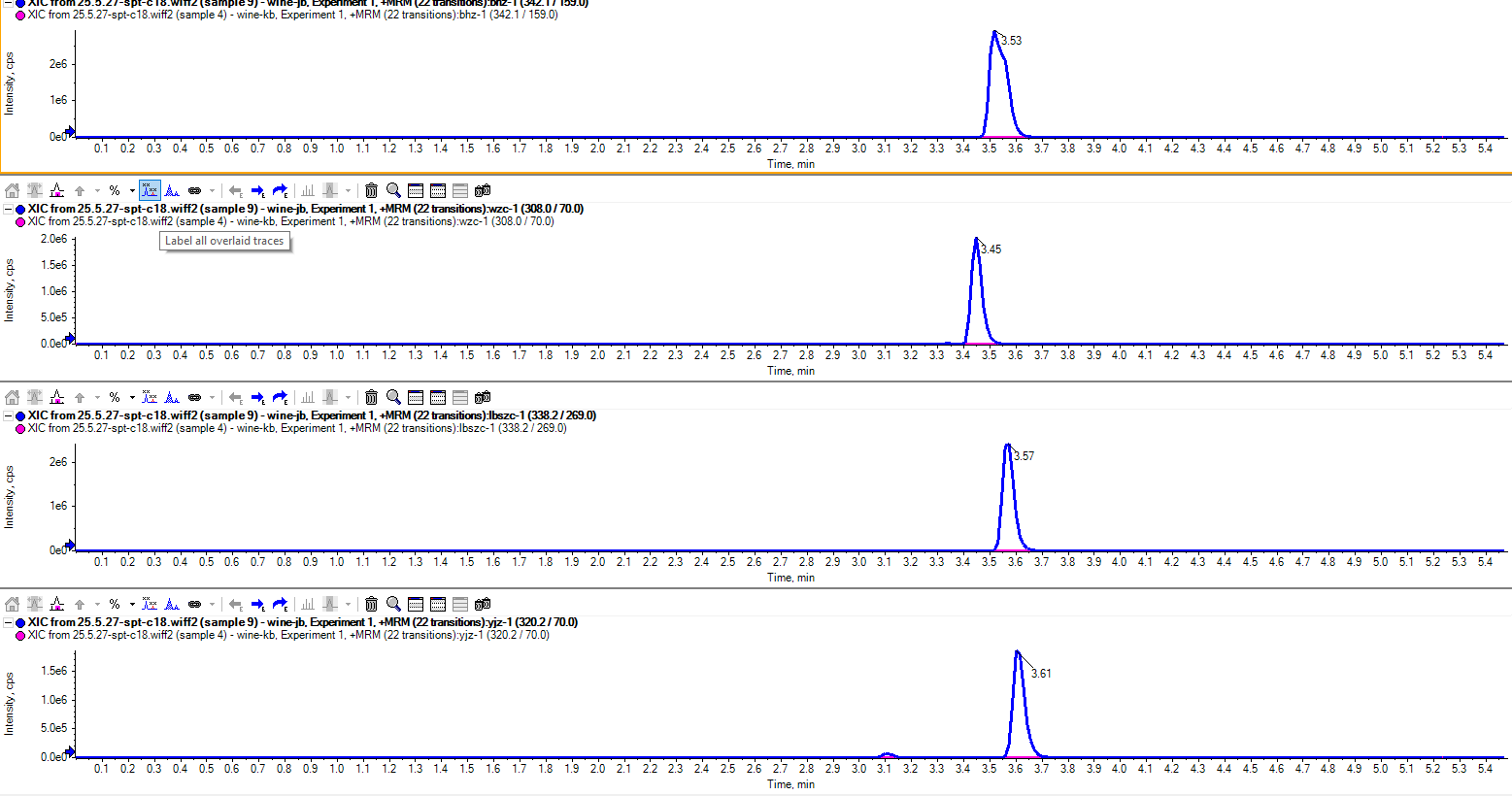


**D**


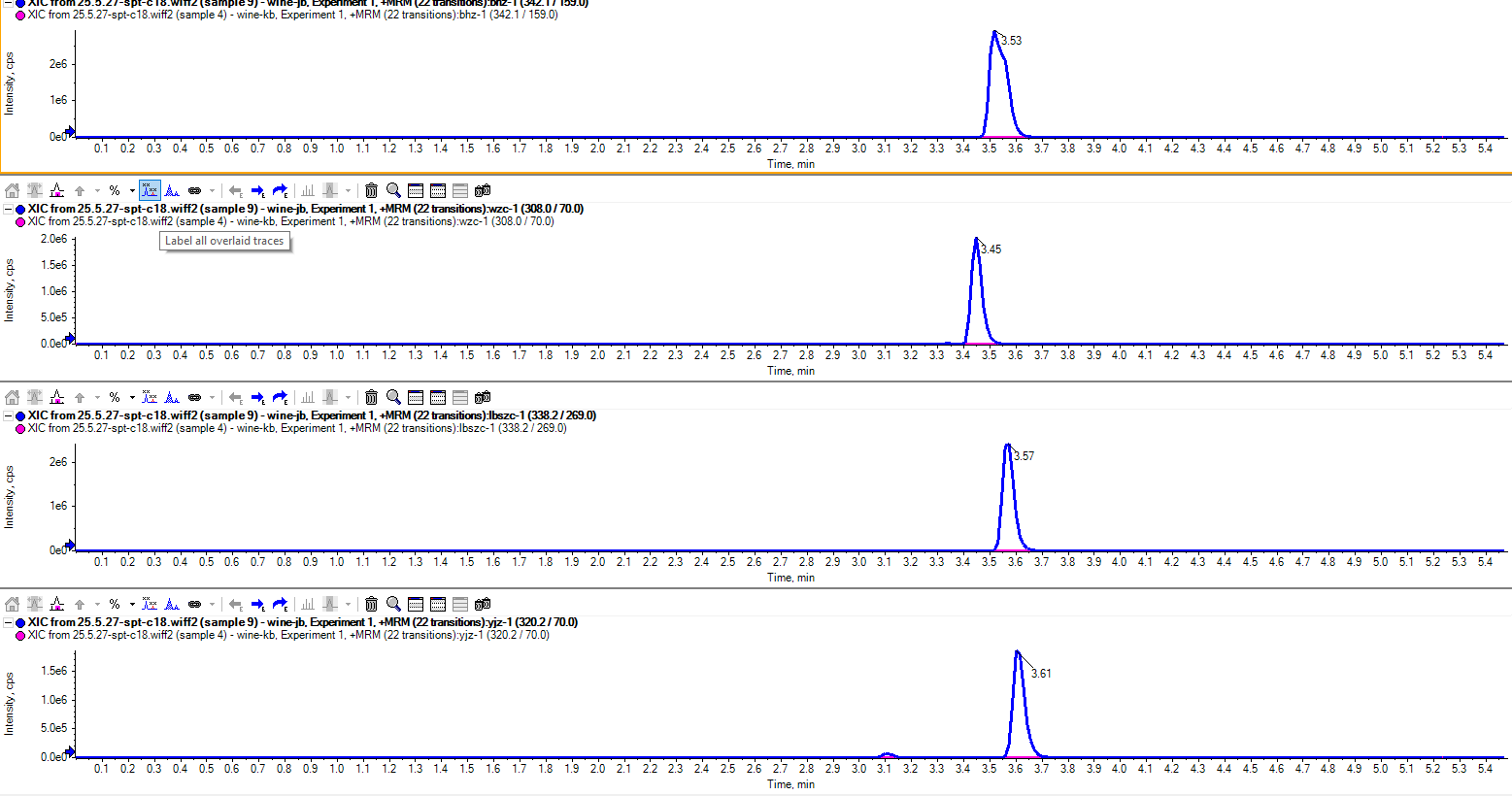


**E**


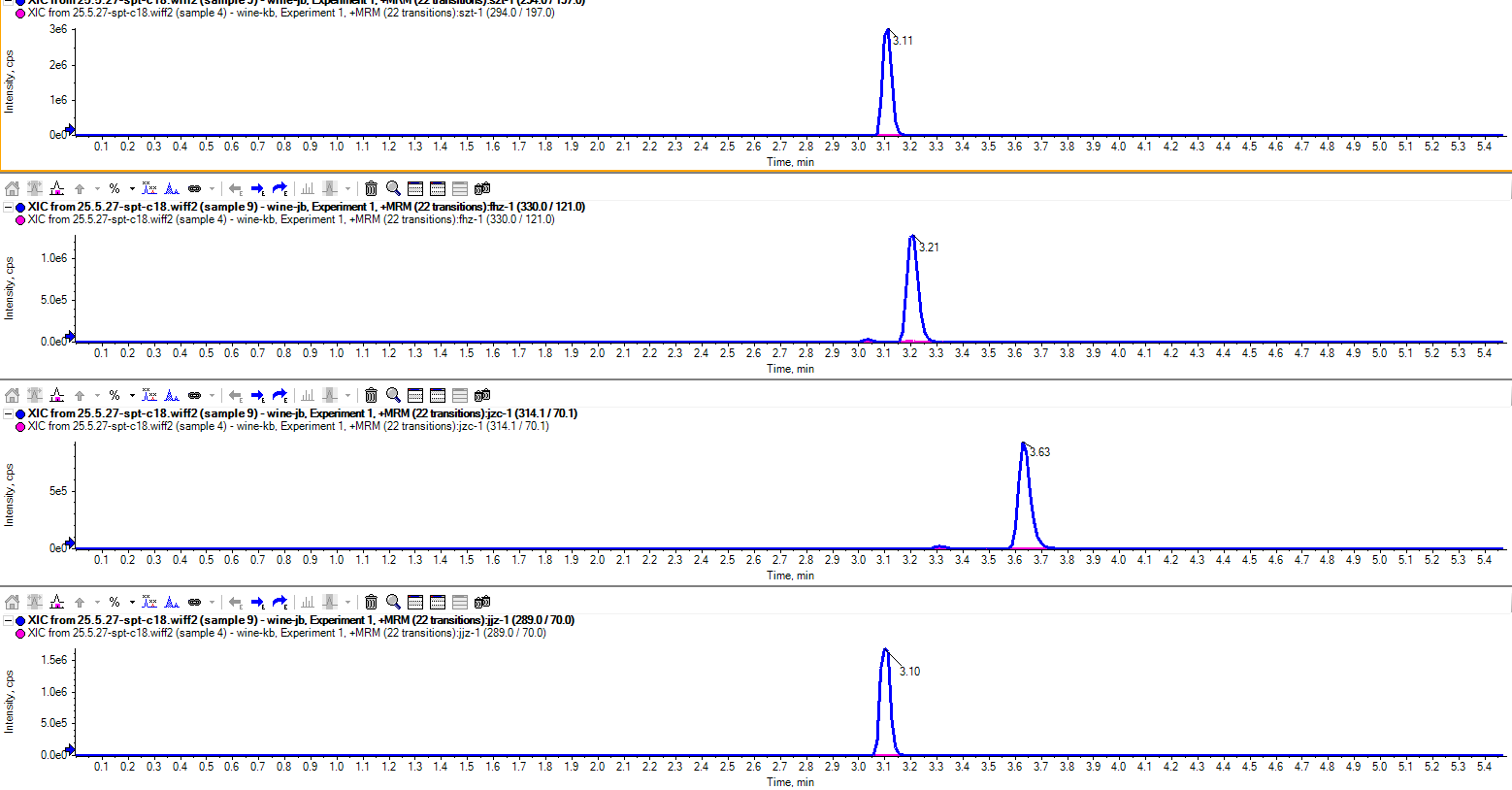


**F**


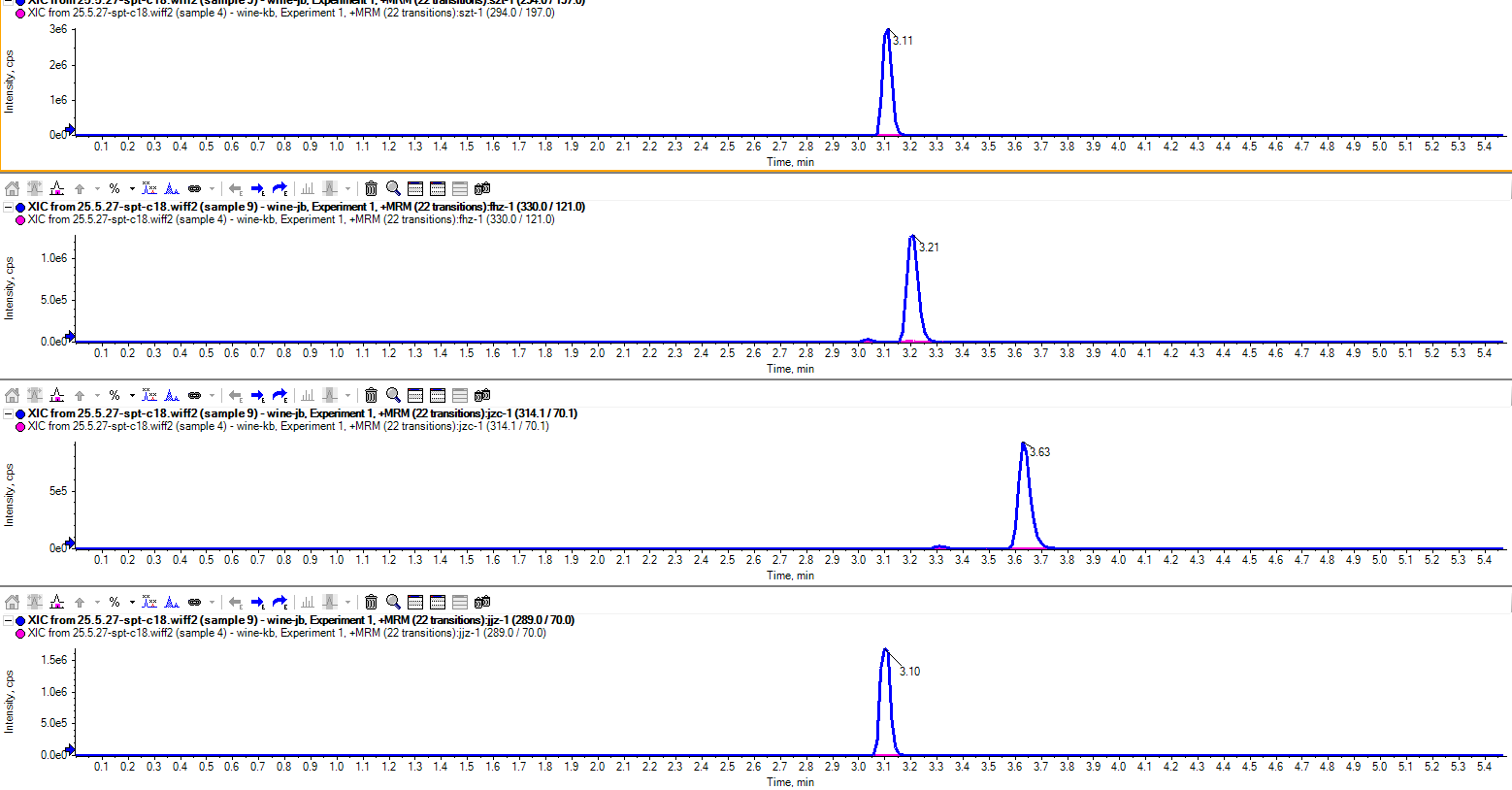


**G**


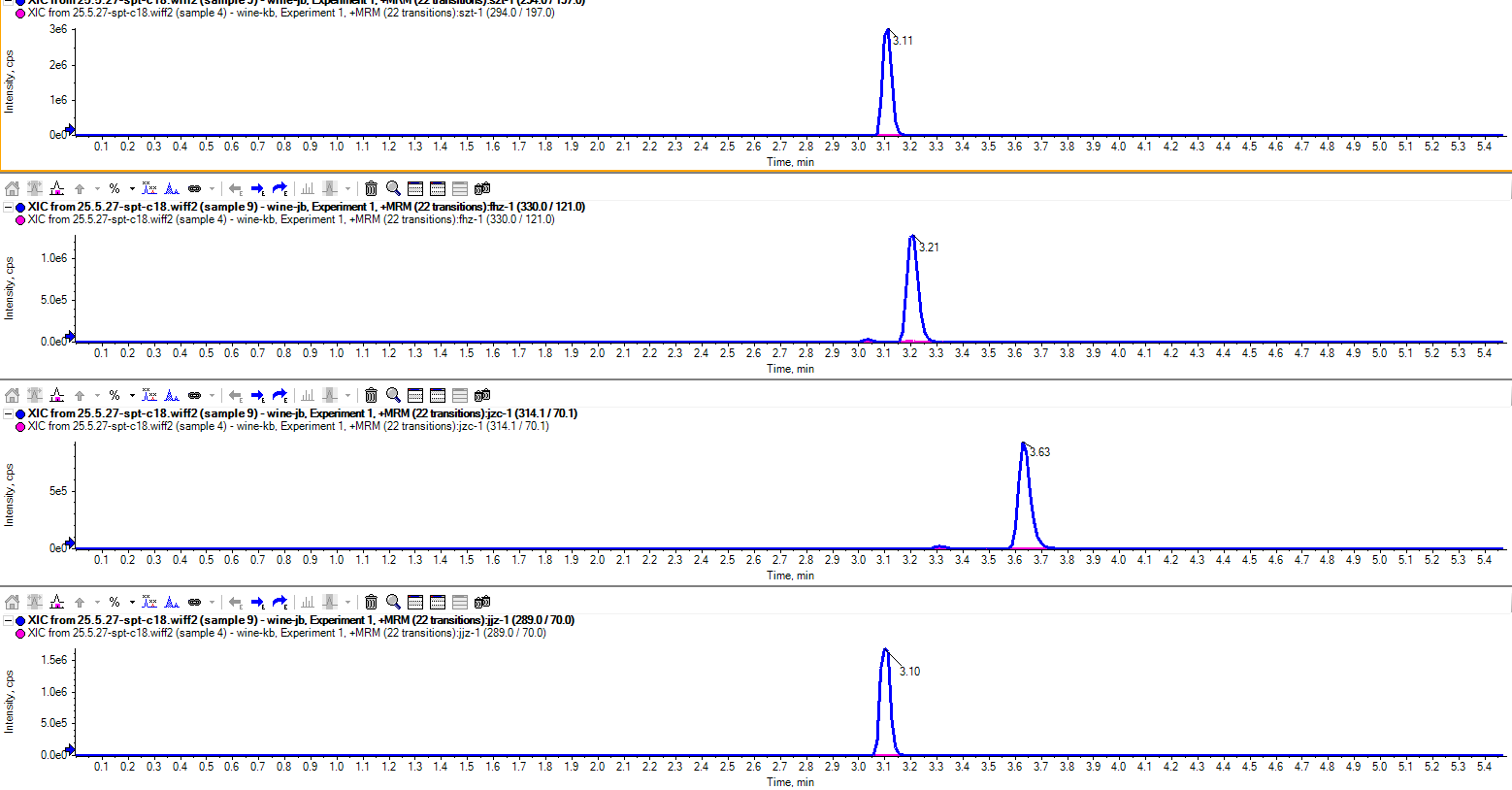


**H**


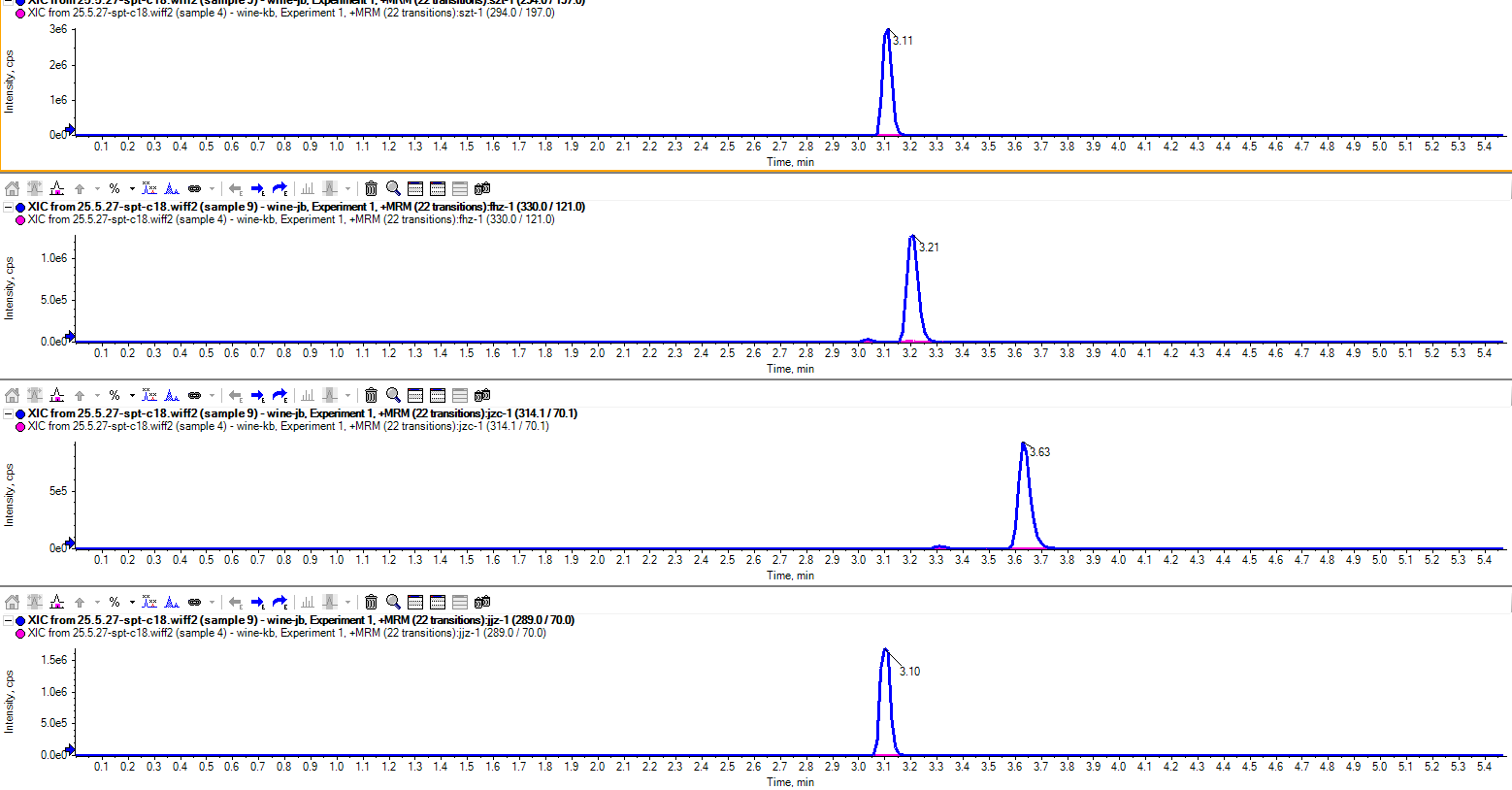


**I**


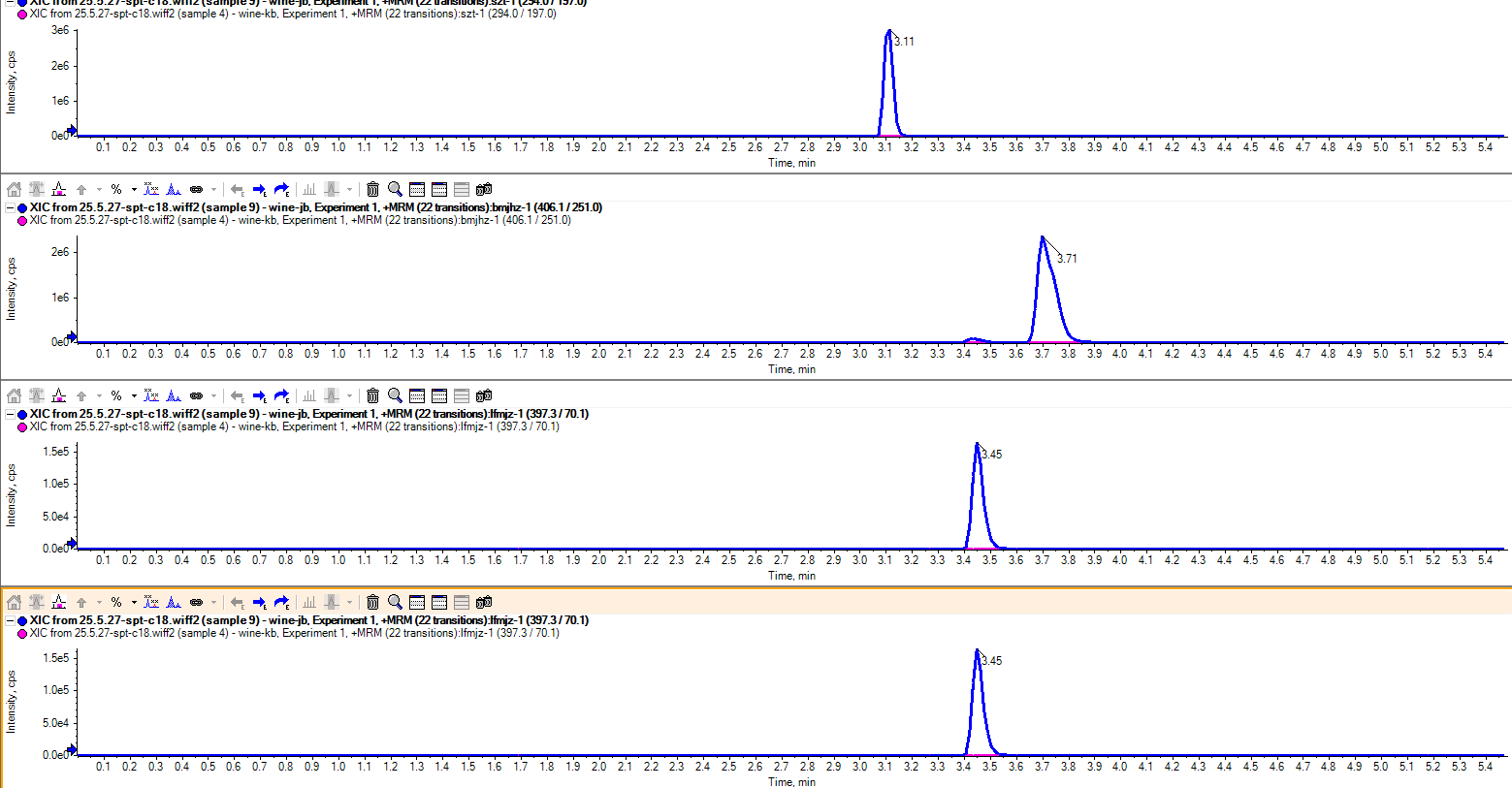


**J**


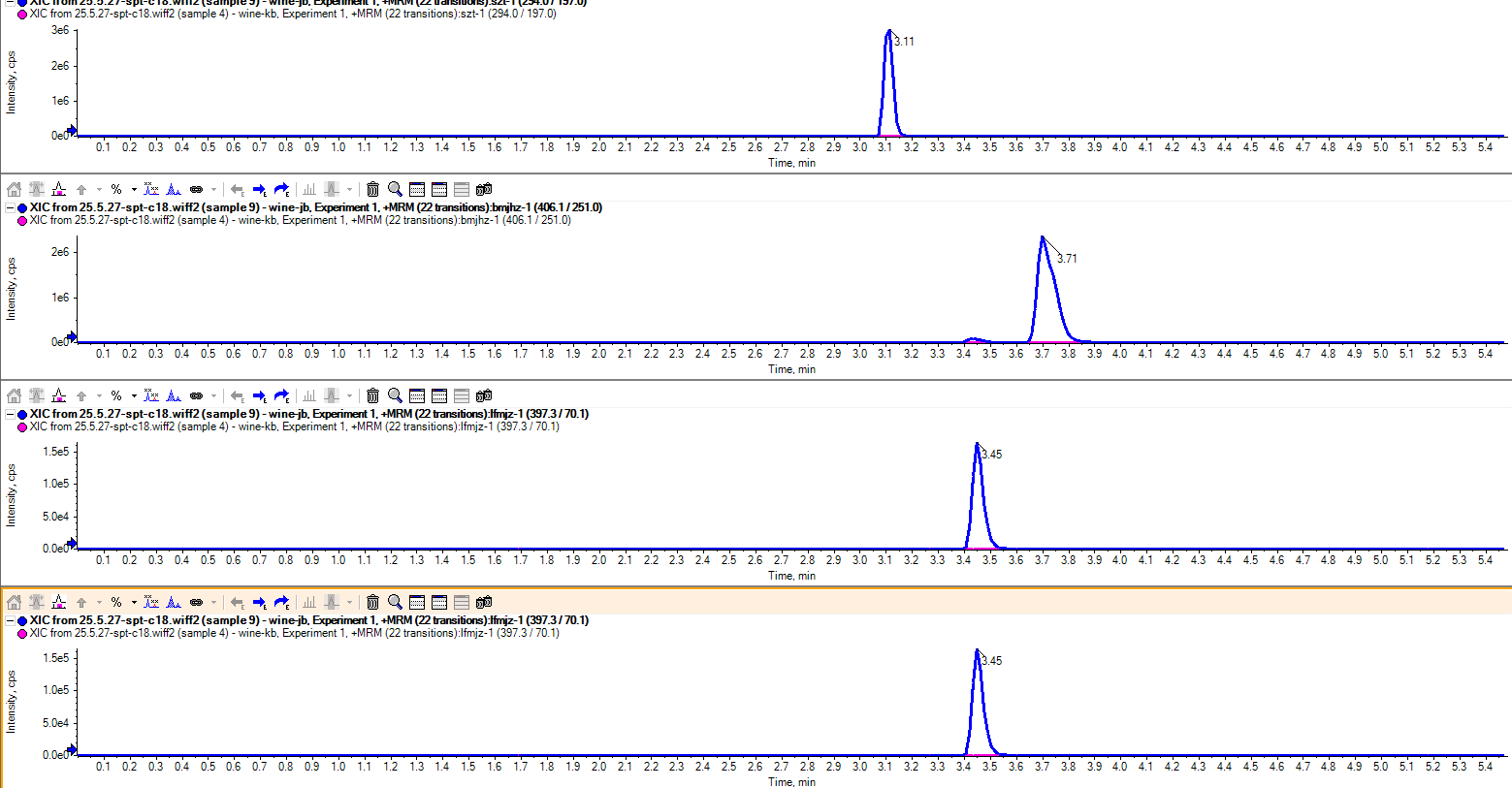


**Fig. S5.** UHPLC-MS/MS chromatograms of ten TFs in wine sample; the blue chromatograms represent the spiked wine, while the pink chromatograms represent the blank wine (A: Propiconazole; B: Tebuconazole; C: Bitertanol; D: Metconazole; E: Triadimefon; F: Epoxiconazole; G: Hexaconazole; H: Myclobutanil; I: Difenoconazole; J: Mefentrifluconazole).

**A**


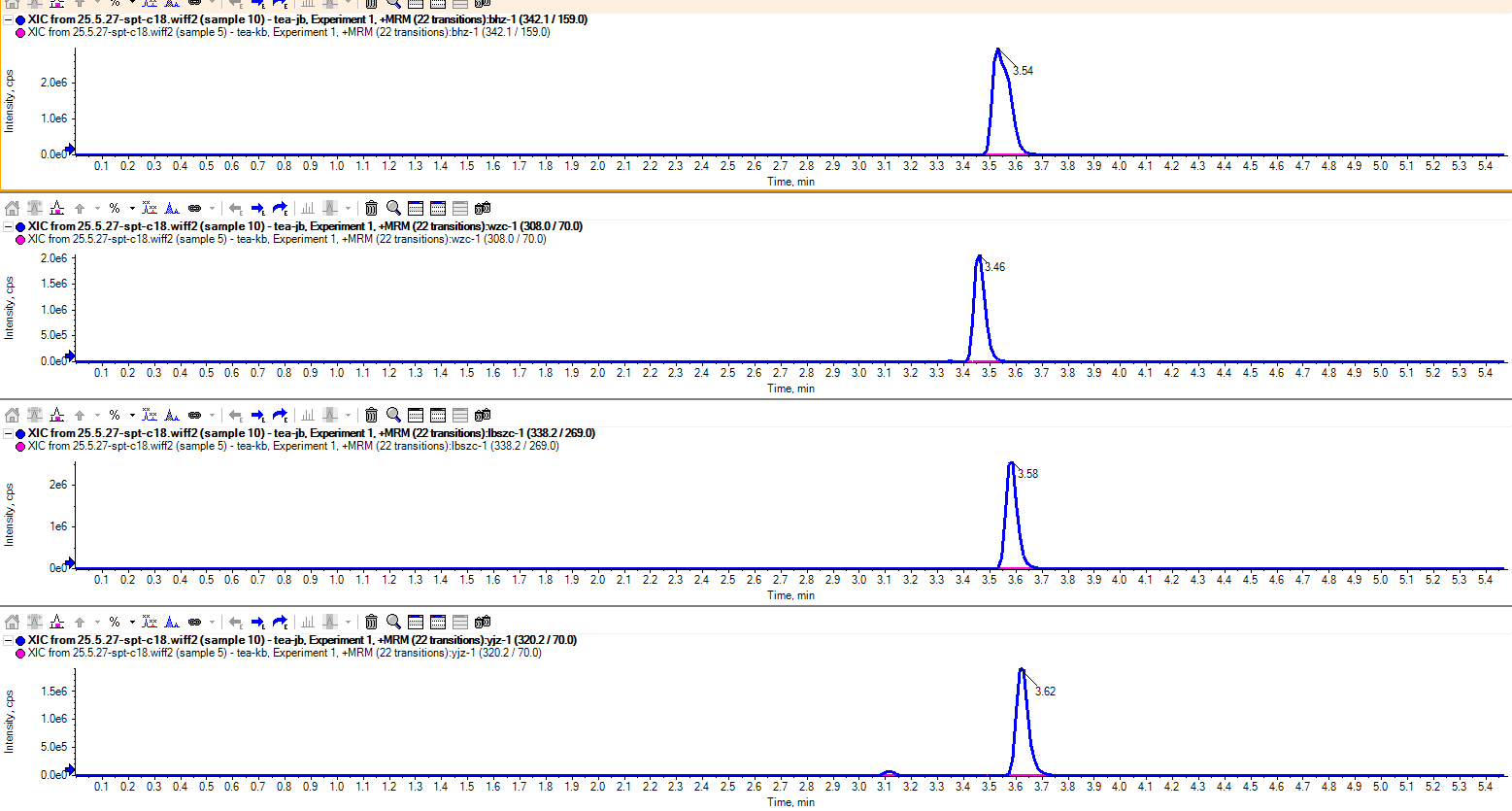


**B**


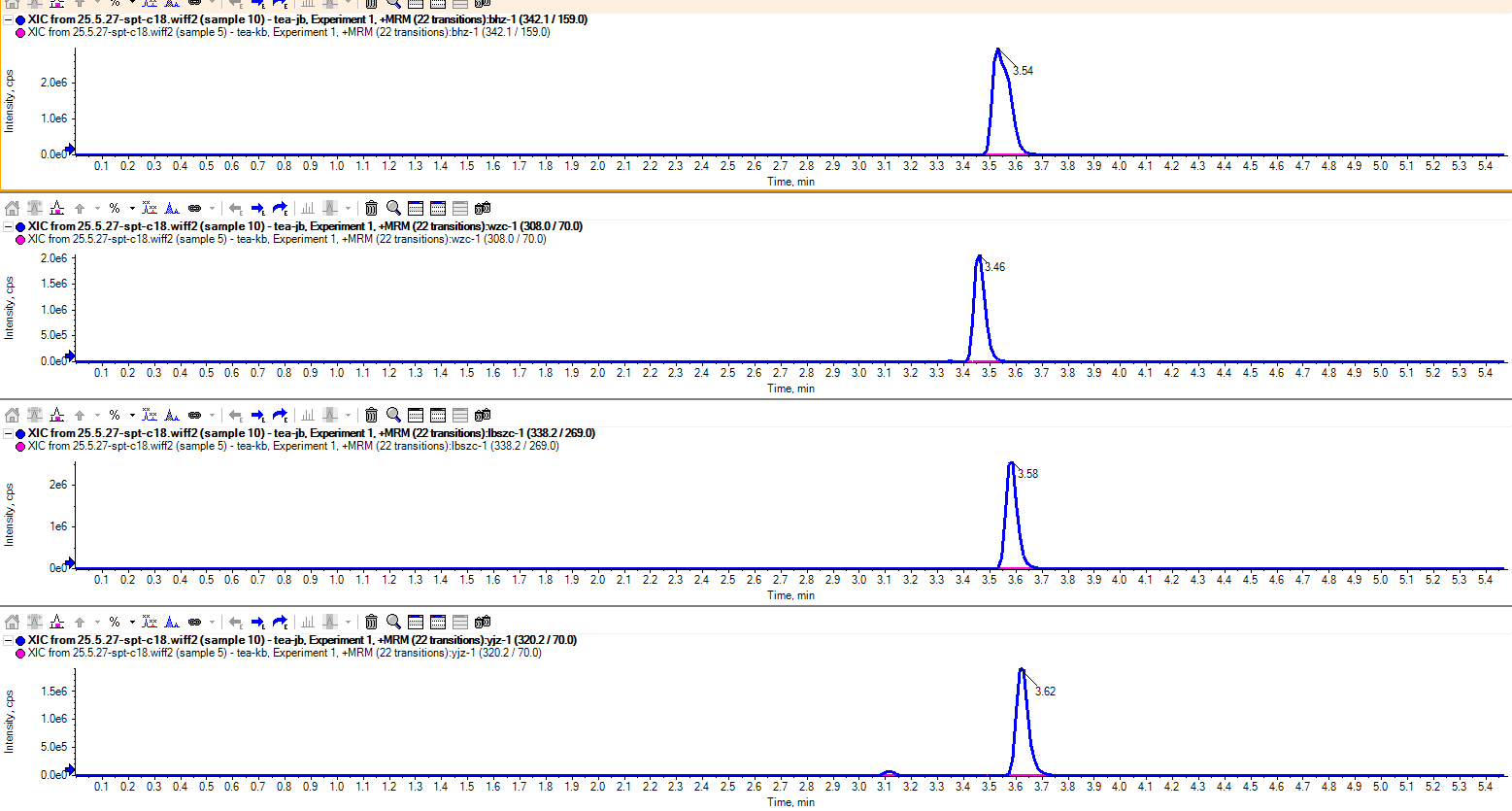


**C**


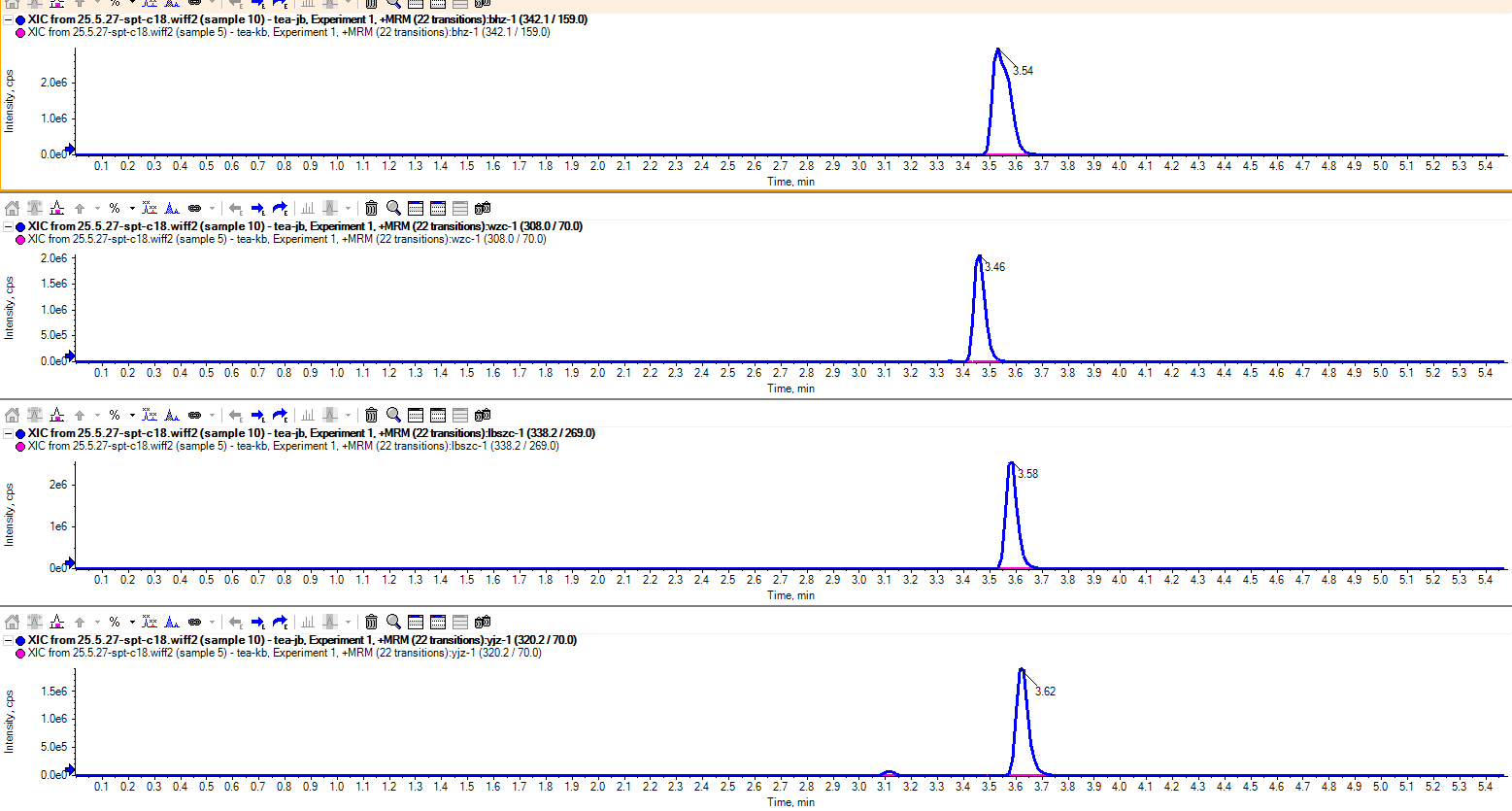


**D**


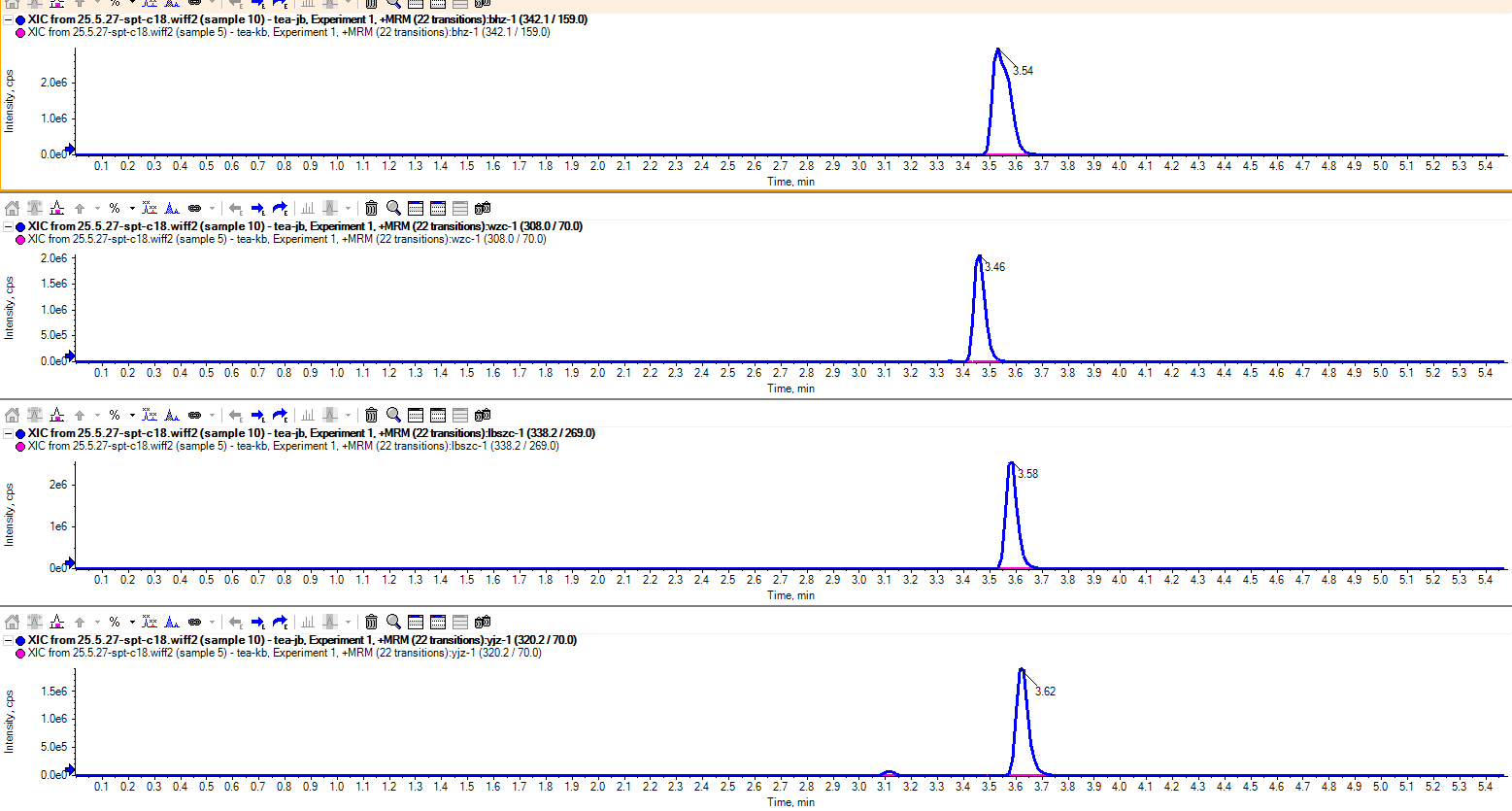


**E**


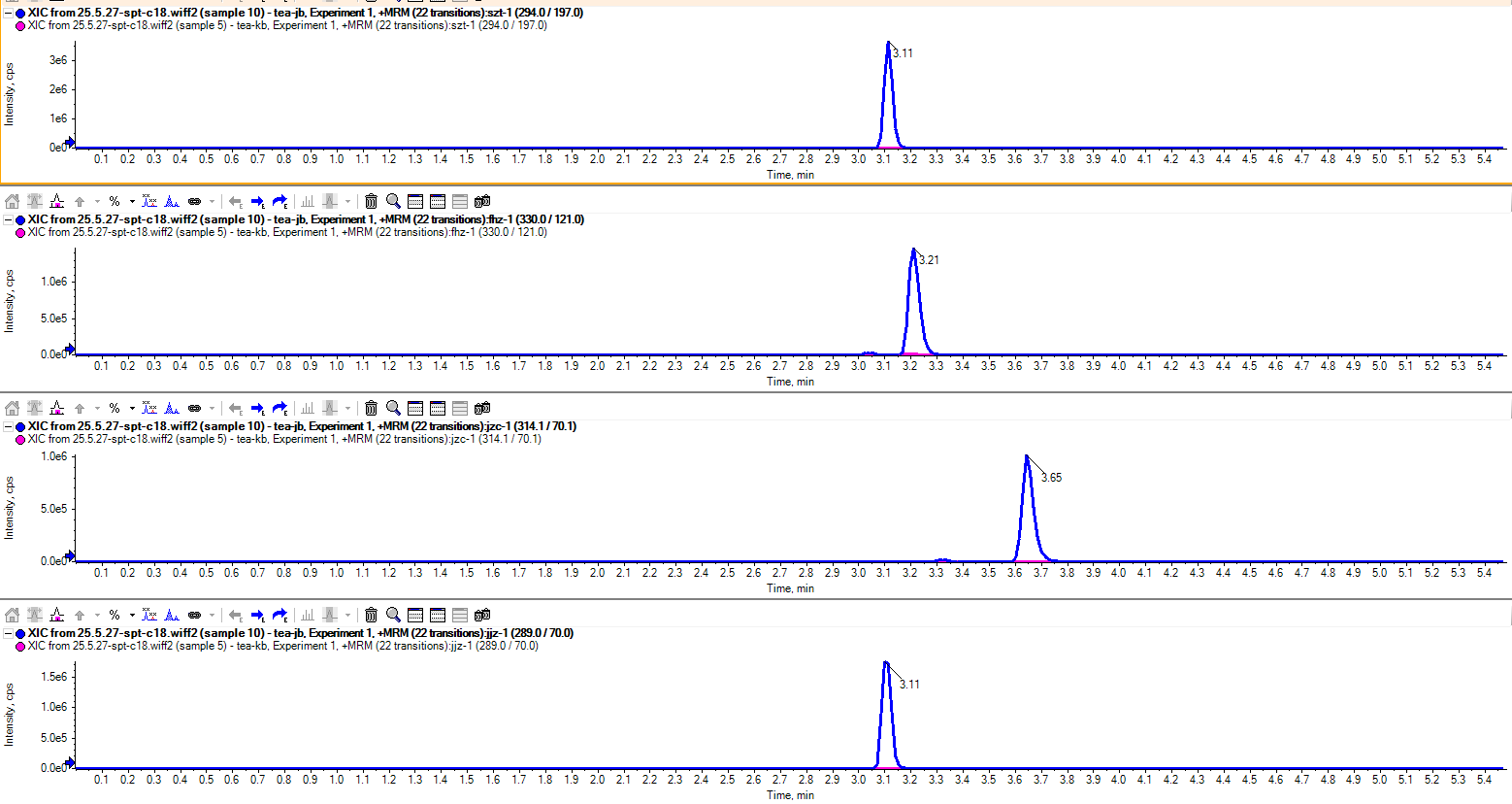


**F**


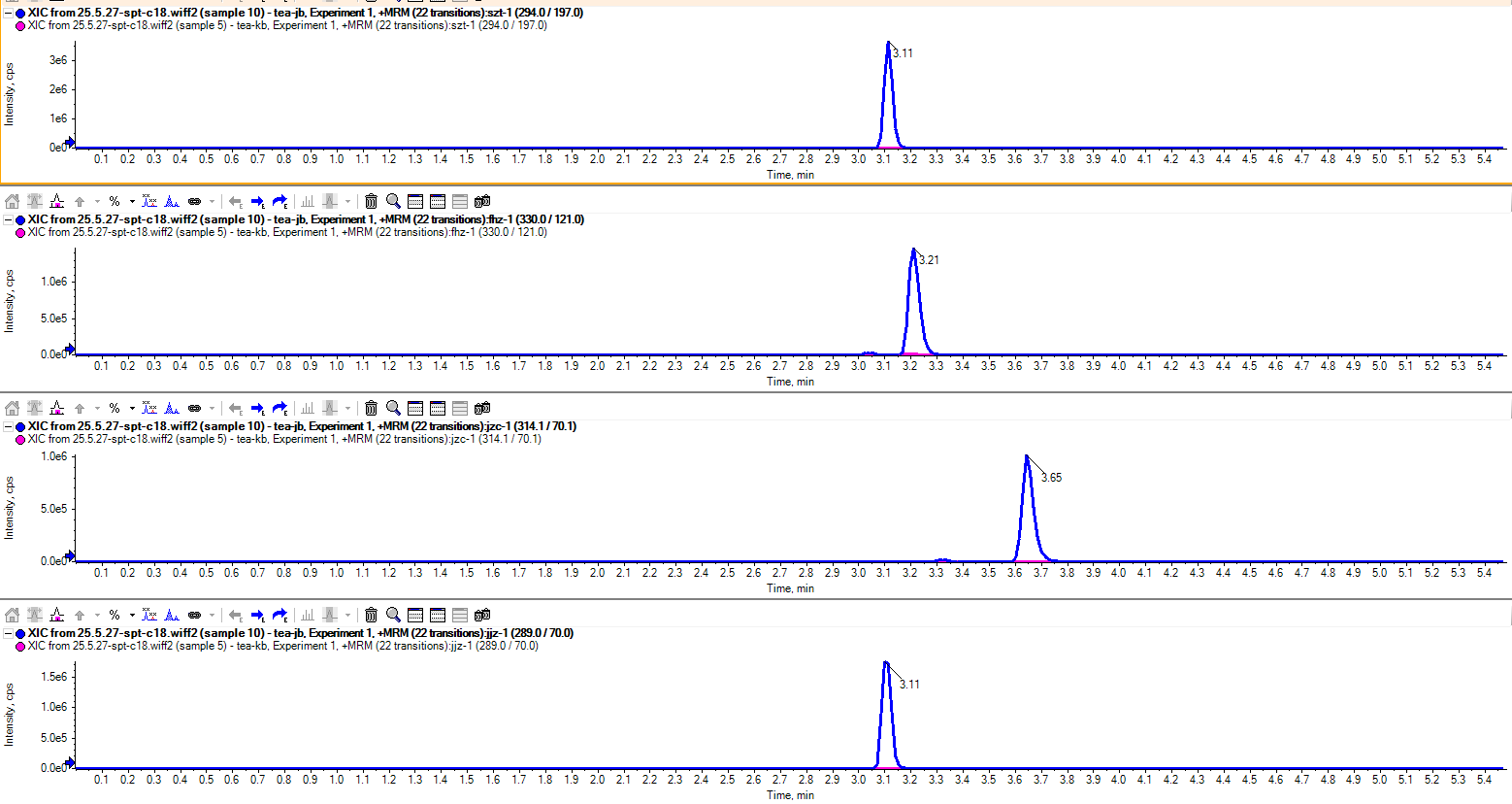


**G**


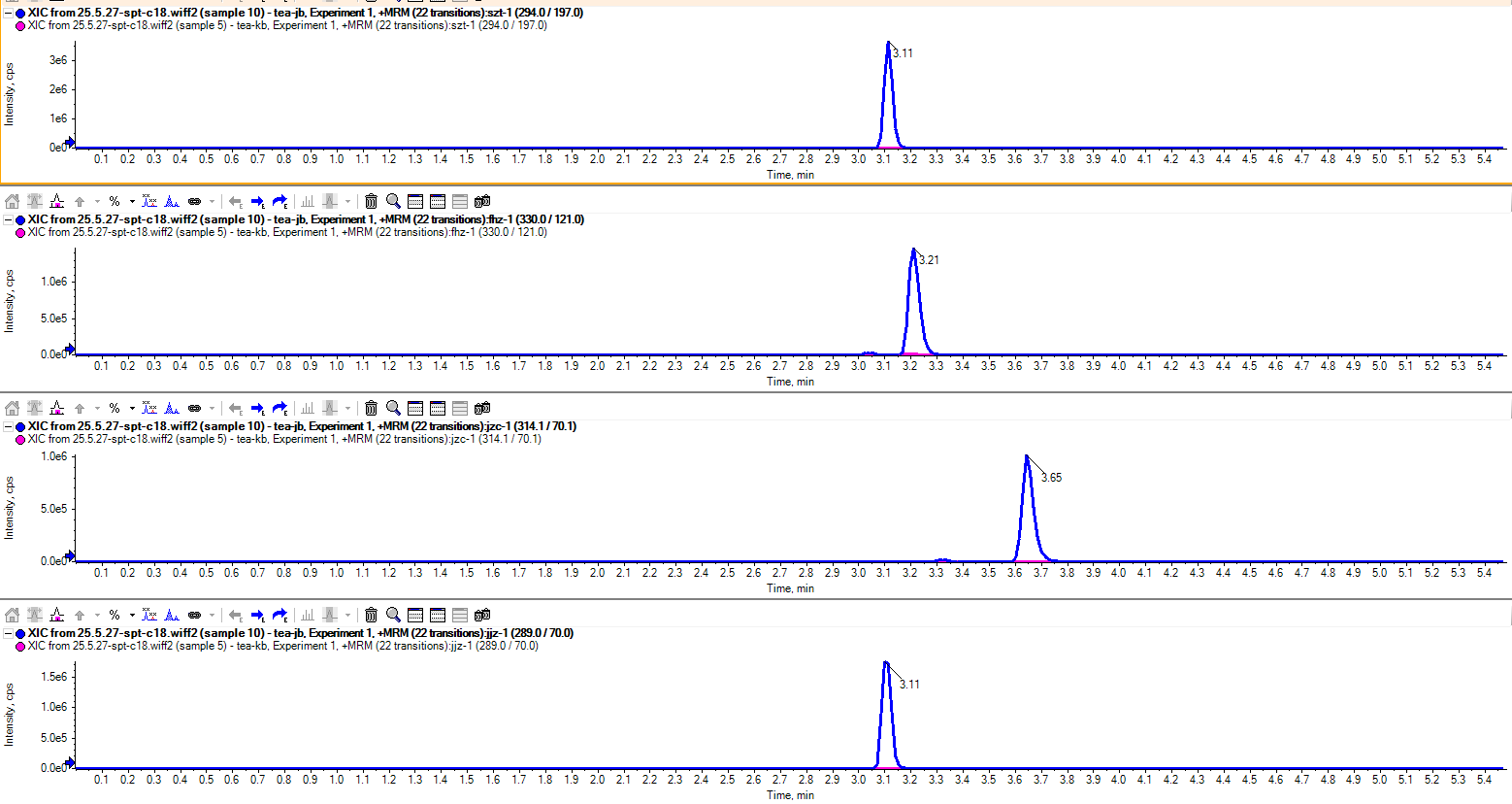


**H**


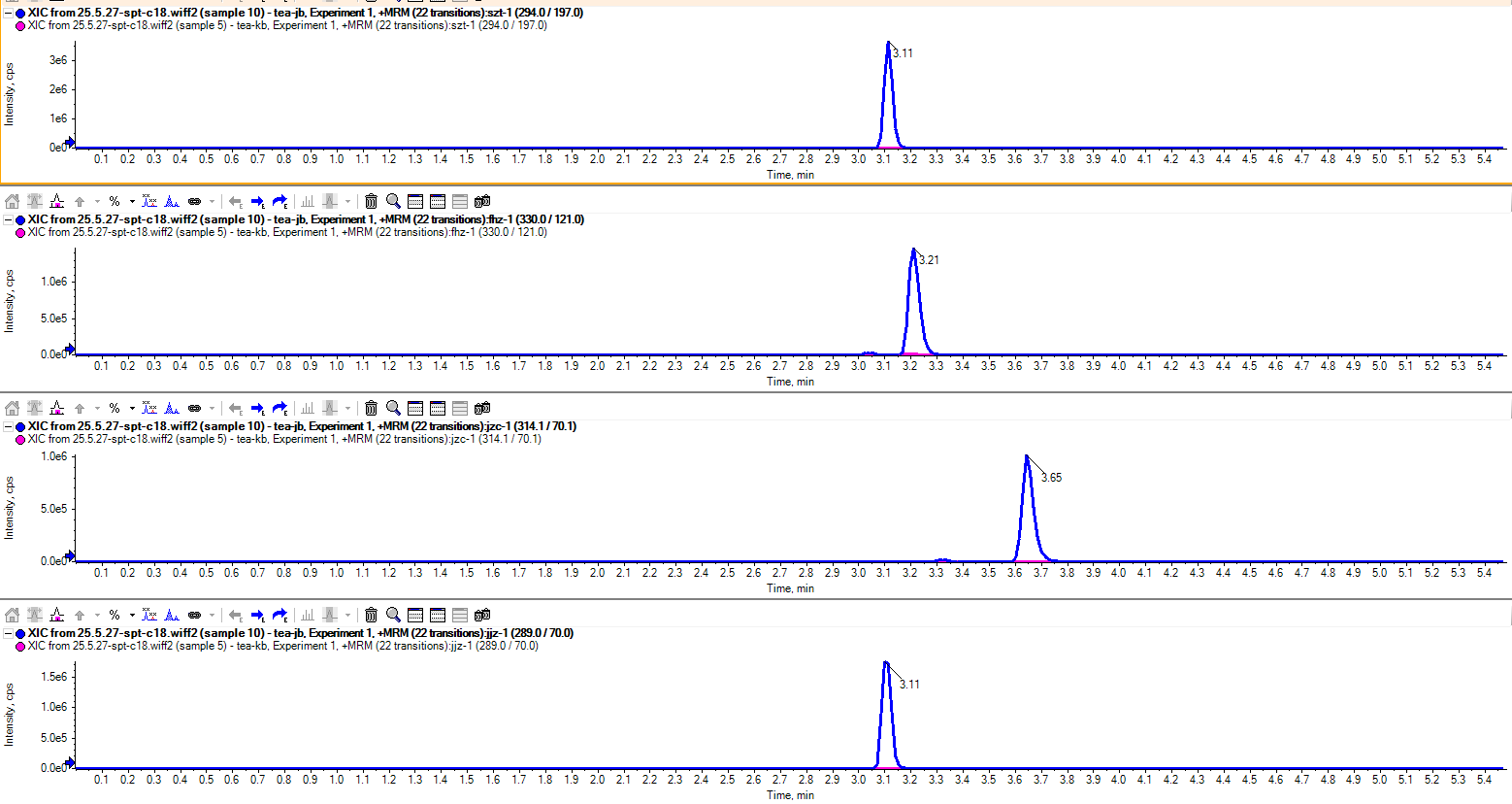


**I**


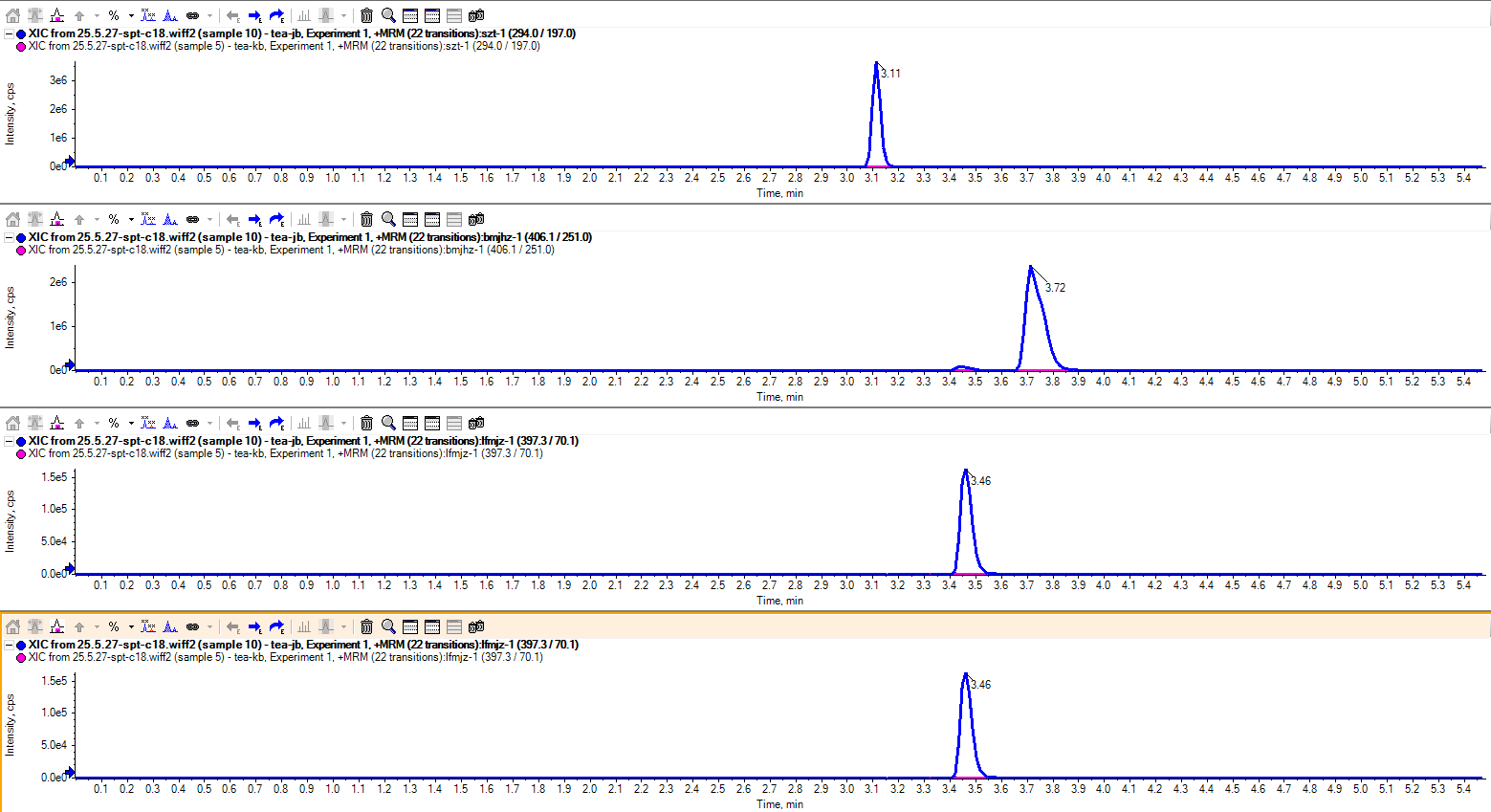


**J**


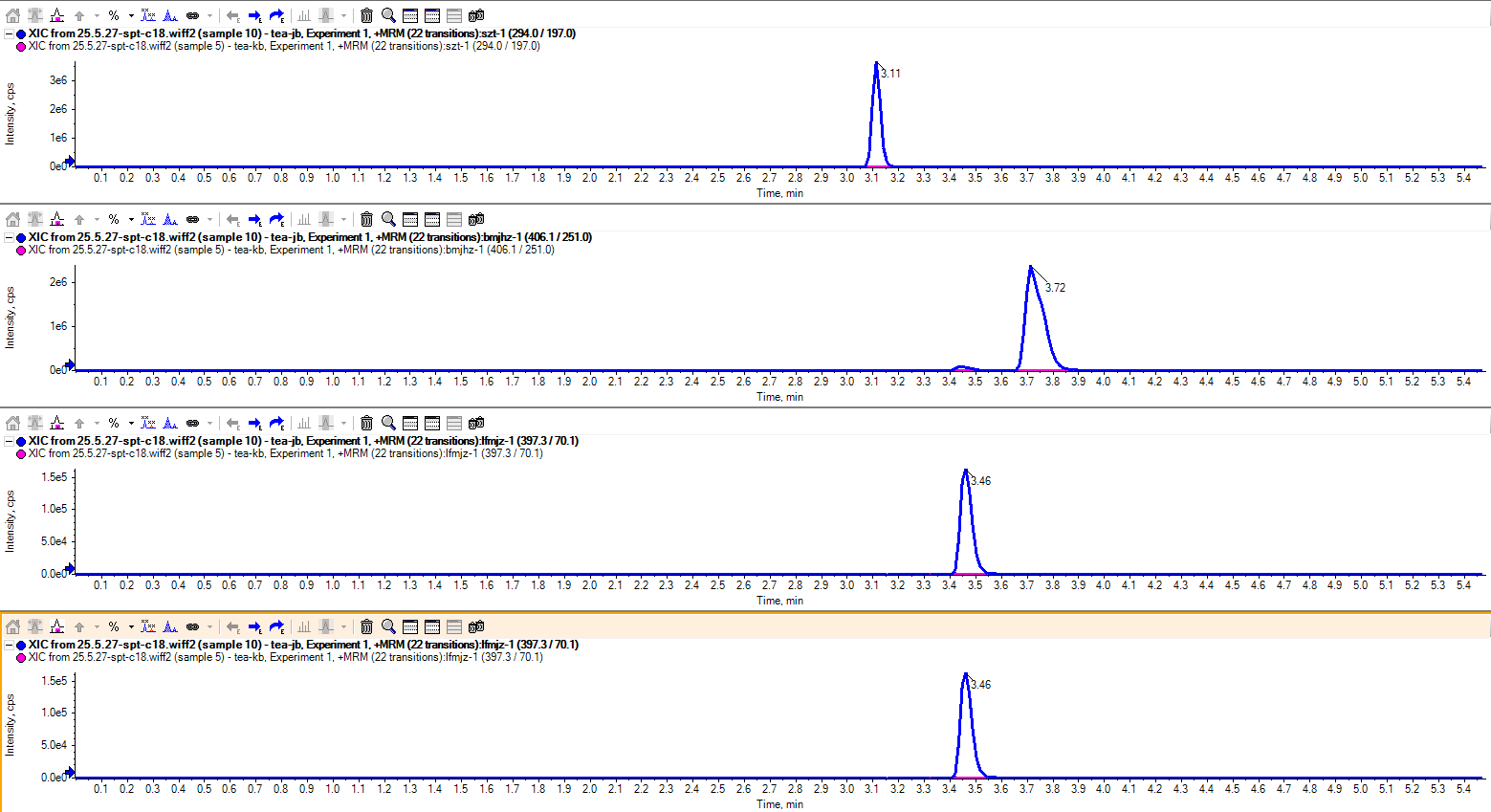


**Fig. S6.** UHPLC-MS/MS chromatograms of ten TFs in tea sample; the blue chromatograms represent the spiked tea, while the pink chromatograms represent the blank tea (A: Propiconazole; B: Tebuconazole; C: Bitertanol; D: Metconazole; E: Triadimefon; F: Epoxiconazole; G: Hexaconazole; H: Myclobutanil; I: Difenoconazole; J: Mefentrifluconazole).

Table S1. Mass spectrometry parameters for Ten TFs

| Triazole fungicide | Precursor ion (m/z) | Qualifier ion  (m/z) | DP  (V) | CE  (V) |
| --- | --- | --- | --- | --- |
| Mefentrifluconazole | 397.3 | 70.1  182.2 | 25 | 66  21 |
| Propiconazole | 342.1 | 159.0  161.0 | 70 | 43  43 |
| Tebuconazole | 308.0 | 70.0  125.0 | 95 | 49  47 |
| Bitertanol | 338.2 | 269.0  251.0 | 20 | 13  16 |
| Metconazole | 320.2 | 70.0  125.0 | 75 | 62  56 |
| Triadimefon | 294.0 | 197.0  225.0 | 70 | 21  17 |
| Epoxiconazole | 330.0 | 121.0  101.0 | 85 | 55  70 |
| Hexaconazole | 314.1 | 70.1  159.0 | 85 | 45  40 |
| Myclobutanil | 289.0 | 70.0  125.0 | 80 | 35  46 |
| Difenoconazole | 406.1 | 251.0  337.0 | 105 | 35  24 |

DP: Declustering potential; CE: Collision energy

Table S2. Detailed information related to sub-items of DLLME-UHPLC-MS/MS based on ComplexMoGAPI assessment.

| Category |  | Evaluation |
| --- | --- | --- |
| Sample preparation | Collection (1) | Off line |
|  | Preservation (2) | None |
|  | Transport (3) | None |
|  | Storage (4) | None |
|  | Type of method (5) | Extraction required |
|  | Scale of extraction (6) | Microextraction |
|  | Solvents/reagents used (7) | Green solvents/reagents |
|  | Additional treatments (8) | None |
| Reagent and solvents | Amount (9) | < 10 mL (< 10 g) |
|  | Health hazard (10) | Moderately toxic; |
|  | Safety hazard (11) | Highest NFPA flammability or instability score of 2 special hazard is used |
| Instrumentation | Energy (12) | 0.37 kWh |
|  | Occupational hazard (13) | Emission of vapors to the atmosphere |
|  | Waste (14) | < 1 mL (< 1 g) |
|  | Waste treatment (15) | No treatment |
|  | Quantification (16) | Yes |
| Yield and conditions | Yield (I) | 70-89 % |
|  | Temperature/time (II) | Room temperature, <1 h |
| Relation to green economy | Number of rules met (III) | 5-6 |
| Reagents and solvents | Health hazard (IVa) | Moderately toxic. NFPA = 2 |
|  | Safety hazard (IVb) | Highest NFPA flammability or instability score is 2 special hazard is involved |
| Instrumentation | Technical setup (Va) | Common setup |
|  | Energy (Vb) | ≤1.5 kWh per sample |
|  | Occupational hazard (Vc) | Emission of vapors to the atmosphere |
| Workup and purification | Workup and purification of the end product (VIa) | None or simple processes |
|  | Purity (VIb) | 97-98 % |
|  | E-factor | 15 |

Table S3. Sub-item scores and related information for DLLME-UHPLC-MS/MS based on SPMS assessment.

| Category | Metric parameter | Value of the parameter | Assigned numeric score | Color score & qualitative rating |
| --- | --- | --- | --- | --- |
| Sample information | Sample amount  (mL or g) | ≤10 | 5 |  |
| Extractant information | Amount of extractant  (mL or g) | 0.183 mL | 12 |  |
|  | Nature of extractant | Natural | 20 |  |
| Procedure information | Number of steps | 3 | 6 |  |
|  | Extraction time  (min) | 5 | 10 |  |
|  | Additional steps after extraction | No additional steps | 10 |  |
|  | Sample throughput | Multiple samples | 3 |  |
| Energy consumption | Dispersion/stir | Without / Manual shake | 0 |  |
|  | Separation | No centrifuge | 2 |  |
|  | Temperature  (℃) | Room temperature | 5 |  |
| Total waste | Waste  (mL or g) | 0.75 mL | 10 |  |
| Reusability of extractant | Reusable | No | 0 |  |
| Global score | | | 83 (out of 95) | |
|  |  |  | 8.42 (out of 10) | |

Table S4. Sub-item scores and related information for DLLME-UHPLC-MS/MS based on GEMAM assessment.

| Sections | Evaluation content | Assessment results | Score | Weight |
| --- | --- | --- | --- | --- |
| Sample information | The position of the analytical device | Ex situ | 0.5 | 0.3 |
|  | Whether the sample is destoryed during sample collection | No | 2 |  |
|  | The range of sample collection | Microextraction | 1.5 |  |
|  | The size of sample | 5.0 | 2.0 |  |
|  | Storage of sample | Under room temperature | 2.0 |  |
|  | The amounts of sample |  | 8.0 |  |
| Reagents  information | Description of the ideal green derivatization | No derivatisation | 1.0 | 0.2 |
|  | The amounts of reagents | 0.75 mL | 1.0 |  |
|  | The score of reagents |  | 2.0 |  |
| Method  information | Analysis parameters | 10 | 2.0 | 0.15 |
|  | Sample throughput (per h) | 40 | 2.46 |  |
|  | Number of main steps in the analysis process | 3 or less | 2.0 |  |
|  | Ratio of the mass of sustainable and renewable materials to the total mass  of materials used | ≥ 75% materials in analysis method  are sustainable or renewable. | 1.5 |  |
|  | Economic benefits of the method | 0.5 | 1.0 |  |
|  | The score of method |  | 8.96 |  |
| Instrument  information | Most energy-intensive | UHPLC-MS/MS | 3.61 | 0.1 |
|  | Energy consumed per sample analyzed | 0.37 kWh |  |  |
|  | Automation of instruments | Automatic | 3.0 |  |
|  | Miniaturization of instruments | No miniaturized | 1.5 |  |
|  | The score of instruments |  | 8.11 |  |
| Waste  information | Waste treatment | No treatmen | 0 | 0.2 |
|  | The amounts of wastes | 0.75 mL | 1.0 |  |
|  | The score of wastes |  | 1.0 |  |
| Operator | Hermetic sealing of analytical process | Emission | 0 | 0.05 |
|  | Noise generating of analytical process | 60.0 dB | 5.0 |  |
|  | The score of operators |  | 5.0 |  |
| Total score |  |  | 5.4 | |

Table S5. Sub-item scores and related information for DLLME-UHPLC-MS/MS based on BAGI assessment.

| Category | Value of the parameter | Color score |
| --- | --- | --- |
| Type of Analysis | Quantitative and Confirmatory |  |
| Multi- or single-element analysis | 10 |  |
| Analytical technique | UHPLC-MS/MS |  |
| Simultaneous sample preparation | 4 |  |
| Sample preparation | DLLME |  |
| Samples per hour | 44 |  |
| Reagents and materials | Common commercially available reagents |  |
| Preconcentration | Required sensitivity is met with one-step preconcentration. |  |
| Degree of automation | Fully automated |  |
| Amount of sample | 5 mL |  |
| Global score |  | 77.5 |
